# Context-specific role of SOX9 in NF-Y mediated gene regulation in colorectal cancer cells

**DOI:** 10.1093/nar/gkv568

**Published:** 2015-06-03

**Authors:** Zhongcheng Shi, Chi-I Chiang, Paul Labhart, Yanling Zhao, Jianhua Yang, Toni-Ann Mistretta, Susan J. Henning, Sankar N. Maity, Yuko Mori-Akiyama

**Affiliations:** 1 Department of Pathology & Immunology, Baylor College of Medicine, Texas Children's Hospital, 1102 Bates Street, Suite FC 830.27, Houston, TX 77030-2399, USA; 2 Active Motif, 1914 Palomar Oaks Way, Suite 150, Carlsbad, CA 92008, USA; 3 Department of Pediatrics, Baylor College of Medicine, Texas Children's Cancer Center, Houston, TX 77030-2399, USA; 4 Department of Medicine, Cell Biology & Physiology, University of North Carolina at Chapel Hill, Chapel Hill, NC 27599-7032, USA; 5 Department of Genitourinary Medical Oncology—Research, Division of Cancer Medicine, University of Texas M. D. Anderson Cancer Center, Houston, TX 77030, USA

## Abstract

Roles for SOX9 have been extensively studied in development and particular emphasis has been placed on SOX9 roles in cell lineage determination in a number of discrete tissues. Aberrant expression of SOX9 in many cancers, including colorectal cancer, suggests roles in these diseases as well and recent studies have suggested tissue- and context-specific roles of SOX9. Our genome wide approach by chromatin immunoprecipitation sequencing (ChIP-seq) in human colorectal cancer cells identified a number of physiological targets of SOX9, including ubiquitously expressed cell cycle regulatory genes, such as *CCNB1* and *CCNB2, CDK1*, and *TOP2A*. These novel high affinity-SOX9 binding peaks precisely overlapped with binding sites for histone-fold NF-Y transcription factor. Furthermore, our data showed that SOX9 is recruited by NF-Y to these promoters of cell cycle regulatory genes and that SOX9 is critical for the full function of NF-Y in activation of the cell cycle genes. Mutagenesis analysis and *in* *vitro* binding assays provided additional evidence to show that SOX9 affinity is through NF-Y and that SOX9 DNA binding domain is not necessary for SOX9 affinity to those target genes. Collectively, our results reveal possibly a context-dependent, non-classical regulatory role for SOX9.

## INTRODUCTION

SOX proteins, high-mobility group, (HMG)-box transcription factors, play crucial roles in embryonic and adult diverse tissues; these include maintenance of stem cell properties, lineage specification and terminal differentiation in a cell-type and tissue-specific manner. In the intestinal epithelium, SOX9 is expressed in the stem/progenitor cells, as well as in the nuclei of terminally differentiated Paneth cells of the small intestinal crypts and tuft cells in the villi and it plays a crucial role in Paneth cell differentiation (1,2). Aberrant expression of SOX9 in some human cancers, including colorectal cancer and in *Apc*(min/+) mouse intestinal adenomas suggest roles of SOX9 in colorectal cancer and to delineate the roles of SOX9 in colorectal cancer has been a focus of some recent studies. However, there have been reports of opposing SOX9 functions, promoting and suppressing of proliferation, which suggest diverse, context-specific functions of SOX9 in proliferation. This has been shown in both developmental and cancer contexts. For instance, in normal chondrocytic CFK2 cells and M12 prostate tumor cells, SOX9 overexpression resulted in decreased proliferation rate and cell cycle arrest in G0/G1 (3,4), while *Sox9* knockdown in rat mesenchymal stem cells (MSCs) resulted in a marked decrease in proliferation rate through delayed S-phase progression and increased nuclear localization of p21 (5). Furthermore, differential roles of SOX9 have been demonstrated in normal intestinal epithelium; low SOX9 expression was associated with enhanced proliferative capacity and high SOX9 expression suppressed proliferation (6). Another study showed that SOX9 expression facilitated growth and proliferation of colorectal cancer cells, whereas inactivation reduced tumorigenicity (7).

To gain insight into SOX9-mediated transcriptional regulation in colorectal cancer cells, we first attempted to identify its physiological targets on a genome-scale using chromatin immunoprecipitation (ChIP) followed by sequencing (ChIP-seq) in human colorectal cancer cells. Our ChIP-seq analysis revealed a large number of SOX9 transcriptional targets in diverse pathways. Interestingly, motif analysis revealed CCAAT, a binding sequence for the heterotrimeric NF-Y transcription factor, as a preferred SOX9 binding sequence, in addition to the previously identified classical consensus motif, ^A^/_T_^A^/_T_CAA^A^/_T_G. Statistical analysis of the ChIP-seq data further revealed that many physiological SOX9 targets through the CCAAT motif are on cell cycle regulatory genes, including the promoters of well-characterized G2/M-specific genes, such as cyclin B1 (*CCNB1*) (8), cyclin B2 (*CCNB2*) (9), cyclin dependent kinase 1 (*CDK1*) (10) and topoisomerase II α (*TOP2A*) (11). The current study revealed that, in colorectal cancer cells, SOX9 is recruited by NF-Y to the target genes and interacts with NF-Y on CCAAT promoter sequences and that SOX9 is necessary for the function of NF-Y in activating expression of some cell-cycle regulatory gene expressions.

## MATERIALS AND METHODS

### Cell culture

HT29 and HCT116 cells, both human colon adenocarcinoma cell lines, were purchased from ATCC and cultured in McCoy's 5A Medium (ATCC, Manassas, VA, USA) with 10% FCS (Lonza, Verviers, Belgium) at 37°C in a 5% CO_2_ atmosphere.

### ChIP-seq

ChIP assays were performed as previously described (12) using an anti-SOX9 antibody (Millipore, Billerica, MA, USA) and an anti-NF-YA antibody (Santa Cruz Biotechnology, Santa Cruz, CA, USA). ChIP-seq experiment was performed from one biological replicate. SOX9 ChIP was subjected to sequencing on Illumina HiSeq 2000. Sequencing of input DNA served as control for peak calling and was also used to generate a control visualization track. Sequences (50-nt reads, single end) were aligned to the human genome (hg19) using BWA algorithm. Only uniquely mapping reads were used in the analysis and duplicate reads were removed. Because the number of final alignments obtained was >2× more for the Input data than for the ChIP data, the Input file was adjusted to the ChIP alignment number by randomly removing excess alignments (downsampling). Aligns were extended *in* *silico* at their 3′- ends to a length of 150 bp, which is the average genomic fragment length in the size-selected library and assigned to 32-nt bins along the genome. The resulting histograms (genomic ‘signal maps’) were stored in BAR files. Peak locations were determined using the MACS algorithm (v1.3.7.1) with a cut-off *P*-value = 1e-7. In an attempt to optimize peak calling and to reduce the calling of false peaks, the mfold parameter was set to 16 (default 32) and the *P*-value cut-off was set to 1e-7. Signal maps and peak locations were used as input data to Active Motif's (Carlsbad, CA, USA) proprietary analysis program, which was used to create Excel spreadsheets containing detailed information on sample comparison, peak metrics, peak locations and gene annotations.

### Bioinformatics analysis

The top 1200 peak sequences (i.e. those with the highest fragment density, from −25 to +25 bp relative to the fragment density peak) were analyzed by MEME (13,14), varying the max width and palindrome requirement settings. Identified motifs were further analyzed with TOMTOM (15) and the presence of SOX9-consensus-like motifs in the 17 847 MACS peak sequences was examined using FIMO (16). Both TOMTOM and FIMO were run with default settings. Human Three publicly accessible NF-YA ChIP-seq data were obtained for bioinformatics analysis. The Encyclopedia of DNA Elements (ENCODE) project was used for genome wide comparison analysis and three data sets from UCSC website (HeLa-S3, Accession number: wgEncodeEH002066; GM12878, Accession number: wgEncodeEH002064; K562, Accession number: wgEncodeEH002021) were used to confirm the alignments of the peaks on key genes. Peak locations were intersected with gene features obtained from the RefSeq database using BEDTools (17) and overlap counts were determined using standard UNIX commands. Tag counts in regions containing either a SOX9 peak or a SOX9 peak overlapping with an NF-YA peak were extracted from the signal maps using the Active Motif pipeline mentioned above and plotted in R (18). Pathway analysis was carried out through the use of QIAGEN's Ingenuity^®^ Pathway Analysis (IPA^®^, QIAGEN Redwood City).

### ChIP-qPCR

ChIP was performed using the ChIP-IT Express Kit (Active Motif) according to the manufacturer's instructions, with minor modifications. Cells were cross-linked with 1% formaldehyde, disrupted with a Dounce homogenizer and the chromatin was isolated. The lysates were sonicated to shear the DNA to an average length of 300–600 bp. Chromatin was immunoprecipitated using an anti-SOX9 (Millipore) antibody or anti-NF-YA antibody (Santa Cruz Biotechnology). ChIP DNAs were purified and ChIP enrichment was quantified by quantitative PCR (qPCR). Fold enrichment was determined by the percent input method. Several key SOX9 and NF-YA peaks identified with ChIP-seq assay were validated on multiple biological samples by qPCR, minimum biological triplicates. Standard deviation (SD) was determined using triplicate measurements. PCR primers were designed to amplify regions spanning the center of peaks obtained from ChIP-seq data and a control primer pair was designed to amplify a region in a gene desert on chromosome 12. Primers used for ChIP-qPCR are listed in Supplementary Table S1.

### RNA extraction and RT-PCR analysis

Total RNA was extracted using Trizol reagent (Life Technologies, Gaithersburg, MD, USA) and treated with RNase-free DNase (Roche, Indianapolis, IN, USA) according to manufacturer's instructions. One microgram of total RNA was converted to cDNA using the Transcriptor First Strand cDNA Synthesis Kit (Roche). Real time qRT-PCR analyses were performed using FastStar Universal SYBR Green Master Mix (Roche) and amplified on the Applied Biosystems 7900HT instrument. Primers used were: CCNB1 (forward, 5′- TACCTATGCTGGTGCCAGTG-3′ and reverse, 5′- CAGATGTTTCCATTGGGCTT-3′), CDK1 (forward, 5′-AAGCCGGGATCTACCATACC -3′ and reverse, 5′-CATGGCTACCACTTGACCTGT -3′). Relative levels of gene expression were analyzed using the 2^ΔΔCt^ method.

### Plasmids and transfection

FLAG-tagged wild-type and HMG-deleted SOX9 constructs were generously provided by Dr Benoit de Crombrugghe (the University of Texas, M. D. Anderson Cancer Center). The FLAG-tagged SOX9–303ΔC construct was derived from the FLAG-tagged wild-type SOX9 construct using primers spanning the sequence encoding the first 303 amino acids of human SOX9. FLAG-SOX9ΔHMG-NLS construct was generated by adding an extra nuclear localization sequence, PKKKRKV, encoded by the sequence CCCAAGAAGAAGCGGAAGGTG, to the C terminus of FLAG-SOX9ΔHMG. The fragment of 0.8 kb human TOP2A promoter was cloned into a pGL3 luciferase vector (Promega, Madison, WI, USA). Mutant human TOP2A promoter was made by 1 bp mutation in the putative SOX9 enriched site (−106 to −102 bp upstream of TSS) using the QuickChange Site-Directed Mutagenesis Kit (Stratagene, La Jolla, CA, USA). 1 × 10^6^ HCT116 cells were transfected with 10 μg of plasmids using X-tremeGENE HP DNA Transfection Reagent (Roche) following the manufacturer's instructions. Cells were collected for the following assays 48 h after transfection.

### Knockdown of *SOX9* and *NFYA*

Short interfering RNA (SiRNA)-mediated *SOX9* knockdown was performed as previously described using *SOX9* siRNA (19). For *NFYA* knockdown, cells were transfected with 75 nM siRNA targeting *NFYA* or control siRNA (Thermo Scientific, Waltham, MA, USA) using DharmaFECT 4 siRNA Transfection Reagent (GE Healthcare, Chalfont St Giles, UK). Cells were harvested 48 h post-transfection for further analysis.

### Immunofluorescence and proximity ligation assay (PLA)

HCT116 cells were transfected with FLAG-SOX9ΔHMG, FLAG-SOX9ΔHMG-NLS or FLAG-SOX9–303 ΔC plasmids. Forty-eight hours later, cells were fixed with 4% paraformaldehyde for 10 min at room temperature and permeabilized with 0.5% Triton X-100 followed by blocking at 37°C for 30 min. Cells were then incubated with primary antibody against FLAG (SCBT), followed by incubation with the Alexa Fluor 555 goat anti-rabbit secondary antibodies (Life Technologies). The cells were mounted in SlowFade^®^ Gold Antifade Reagent with DAPI (Life Technologies) and the red fluorescence was visualized under a fluorescence microscope (Nikon). The interaction between SOX9 and NF-YA was demonstrated by *in* *situ* proximity ligation assay (PLA) using a Duolink *in* *situ* red starter kit (Sigma-Aldrich, Louis, MO, USA) according to the manufacturer's instructions. Briefly, HCT116 and HT29 cells were fixed in 4% paraformaldehyde for 10 min at room temperature and permeabilized with 0.5% Triton X-100 followed by blocking at 37°C for 30 min. Cells were then incubated with primary antibodies against SOX9 (1:500), NF-YA (1:500) or control mouse IgG (1:500), followed by washing and incubation with the secondary antibodies conjugated to PLA probes. After ligation and amplification, the red fluorescence indicating the interaction between SOX9 and NF-YA was visualized under a fluorescence microscope (Nikon).

### Western blotting and immunoprecipitation

HCT116 cells and HT-29 cells were lysed in modified RIPA buffer (1% Nonidet P-40, 0.1% sodium deoxycholate, 150 mm NaCl and 1 mm ethylenediaminetetraacetic acid (EDTA) in 50 mm Tris–HCl, pH 7.5) supplemented with 1× protease inhibitor mixture (Sigma-Aldrich). Solubilized proteins were separated by electrophoresis through 10% Tris-glycine gels and transferred onto PVDF membranes. Primary antibodies used for western analysis were rabbit polyclonal anti-SOX9 (Millipore), mouse monoclonal anti-NF-YA (Santa Cruz Biotechnology) and mouse monoclonal anti-β-actin (Santa Cruz Biotechnology). Secondary antibodies used were horseradish peroxidase-conjugated goat anti-mouse and goat anti-rabbit (Santa Cruz Biotechnology). For co-immunoprecipitation, cells were lysed in RIPA buffer and immunoprecipitated overnight at 4°C with 2 μg antibody and 20 μl protein-A/G agarose beads (Santa Cruz Biotechnology). Bead-bound complexes were washed, eluted and visualized by western blotting.

### Pull-down assays

Nuclear extracts were obtained from HCT116 cells. A total of 100 μg of nuclear proteins were mixed with 2 μg of biotin-labeled double-stranded TOP2A oligos (wild-type TOP2A oligo: 5′ biotin-TCCCGCCTCCCTAACCTGATTGGTTTATTCAAACAAACC; mutated TOP2A oligo: 5′ biotin-TCCCGCCTCCCTAACCTGTTTGGTTTATTCAAACAAACC) and 5 μg of polyIC in 1× binding buffer (5× binding buffer: 75 mM Hepes PH7.9, 400 mM NaCl, 75 mM KCl, 0.1 mM EDTA, 5 mM dithiothreitol (DTT), 5 mM phenylmethanesulfonylfluoride (PMSF), and 15% glycerol) for 60 min on ice. Then 40 μl of magnetic beads (Dynabead M-280 streptavidin) were added into the complexes of reactions followed by rotation at 4°C for 30 min. Beads were washed three times with 500 μl of 1× binding buffer. The associated proteins to the biotin-labeled oligos were detected by western blot using either an anti-NF-YA or an anti-SOX9 antibody.

### Electrophoretic mobility shift assay (EMSA)

Electrophoretic mobility shift assay (EMSA) was performed with double-stranded, biotin-labeled oligonucleotides using the Gelshift Chemiluminescent EMSA Kit (Active Motif) according to the manufacturer's instruction. Recombinant human SOX9 protein (OriGene) and/or unlabeled oligonucleotides (cold probes) were added to the biotin-labeled oligonucleotides. The binding reactions were resolved on a non-denaturing polyacrylamide gel and transferred to a nylon membrane, followed by crosslink using UV cross-linker (Stratagene). The membrane was incubated with HRP conjugated streptavidin. The protein–DNA bindings were detected using SuperSignal West Femto Chemiluminescent Substrate (Thermo Scientific).

### Luciferase reporter activity assays

HCT116 cells were plated in 24-well plate at a density of 4 × 10^4^ cells/well and transfected 24 h later with 500 ng of pGL3 empty vector, 500 ng of human TOP2A wild-type or mutant TOP2A plasmids, together with 2 ng of the TK standardization vector used to normalize the transfection efficiency. Luciferase activity was measured 48 h after the transfection using a Dual Luciferase Reporter Assay system (Promega).

### Statistical analysis

Results are expressed as mean ± SD. Differences between two groups were analyzed using an unpaired Student's *t*- test and a *P* < 0.05 was considered significant. (* *P* < 0.05; ** *P* < 0.01 and *** *P* < 0.001).

## RESULTS

### Identification of SOX9 interaction sites in the genome of human colorectal cancer cells

**Figure 1.**
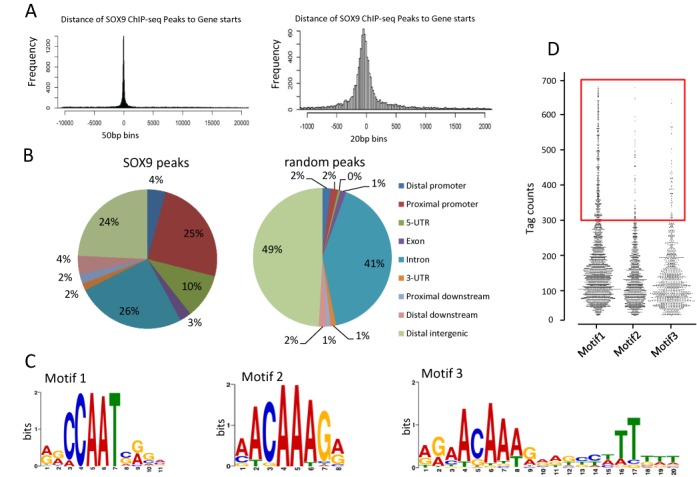
ChIP-seq analysis of SOX9 binding sites in HT29 cells. (**A**) Distribution of SOX9 binding sites relative to nearest TSSs. Histograms were generated from the putative SOX9 binding regions. The left plot (50-bp bins) shows that SOX9- binding sites are enriched symmetrically around TSSs. The right plot (20-bp bins) shows the preferential location between 0 and −100 bp upstream of TSSs. The x-axes represent relative distance (bp) from the TSS. The y-axes represent the frequency of peaks in the whole genome. (**B**) Distribution of SOX9 binding site location relative to RefSeq genes. Locations of binding sites are divided into distal promoter (−1 to −3 kb upstream of TSS), proximal promoter (−1 to 0 kb), 5′ UTR, exon, intron, 3′ UTR, proximal downstream (0–1 kb downstream of TSS), distal downstream (1 to 3 kb downstream of TSS) and distal intergenic (>3 kb from genes). (**C**) Motif analysis from SOX9 ChIP-seq. With identification of statistically over-represented sequences present in ChIP-seq binding loci. A *de novo* putative binding motif was denoted Motif 1, in addition to previously identified consensus motifs, Motif 2 and 3. (**D**) Comparison of tag counts of Motif 1, 2 and 3 genes are demonstrated by the frequency distribution. The red box indicates genes with more than 300 tags.

Aberrant expression of SOX9 in various cancers suggests a role of SOX9 in these diseases. The current study aimed to give insight into roles of SOX9 as a transcription factor by identifying SOX9 target genes in the genome of human colorectal cancer cells. We first carried out ChIP-seq analysis using an antibody specific to SOX9 in HT-29 cells, which express endogenous SOX9 at a considerably high level. MACS peak finding (20) using the default cut-off of *P*-value = 1e-7 identified 17 847 peaks of SOX9 binding enrichment. The empirical false discovery rate, calculated by MACS with a ChIP input sample swap, was 0.01%. For each peak, the distance to RefSeq transcription start site(s) (TSSs) was determined. Figure 1A (left) shows that SOX9 binding sites have a strong preference for TSSs (distance = 0). Figure 1A (right) demonstrates, at higher resolution, that the majority of SOX9 binding sites are located just upstream of the TSS. We also annotated the MACS SOX9 peak locations to RefSeq gene features. As a control, an equal number (17 847) of random genomic locations (generated by sampling input tag locations) were annotated in the same way (Figure 1B). The results demonstrated that SOX9 binding sites are significantly enriched in proximal promoters (within 1 kb upstream of TSS; 25% versus 2%) and 5′-UTRs (10% versus 0.2%), whereas they are underrepresented in intronic and intergenic regions relative to random distribution, despite the high absolute percentages of peaks in these features. Repetitive sequences are known to be many transcription factors binding sites, however, the ‘depletion’ of repeat masked regions in SOX9 peaks was observed (negative log2 values). This indicated that SOX9 peaks are actually underrepresented in repeat masked regions (2474 versus 7820), while simple repeats have close to random occurrence (311 versus 298 hits). It is probably the case that the under-representation of SOX9 peaks in repeat masked regions cannot simply be explained by a difficulty of mapping reads to such regions, as the random peak control was generated based on a random sampling of mapped input reads; in addition, the majority of 50-nt reads are readily mapped to unique locations even in repeat masked regions (Supplementary Figure S1). The data shown in Figure 1A and B thus suggest that in colorectal cancer cells, SOX9 is likely to bind mainly to the proximal promoters of target genes and to some extent through 5′ UTR regions. This is consistent with previous reports by others, including SOX9 ChIP-on-chip analysis of chondrocytes (21) and embryonic gonads (22) and SOX9 ChIP-seq analysis of hair follicle stem cells (23). Several previous studies have identified the sequence ^A^/_T_^A^/_T_CAA^A^/_T_G as the canonical consensus motif for SOX9 (21,24). To confirm whether SOX9 also binds to DNA through this motif in colorectal cancer cells, we used the motif discovery tool MEME. The top 1200 peak sequences (50 bp; ±25 bp relative to summits) were submitted to MEME using different parameters. The three main motifs identified are shown in Figure 1C and are referred to as Motif 1, 2 and 3 throughout in this study. Motif 2 is a perfect match to the classical SOX9 consensus sequence. Motif 3 is an inverted repeat of the 7-bp core consensus sequence separated by 4 bp. This dimeric binding site has been reported to be required for SOX9 dimerization to regulate gene expression in chondrogenesis (25). To our surprise, Motif 1, which has not previously been reported as a SOX9 binding motif, was the most significant motif in all runs (unless searching for palindromes only), with even relatively higher affinity for SOX9, as shown by the number of tags compared to Motif 2 and/or 3 (Figure 1D).

### SOX9 binding sites overlap NF-YA sites in genome of human colorectal cancer cells

**Figure 2.**
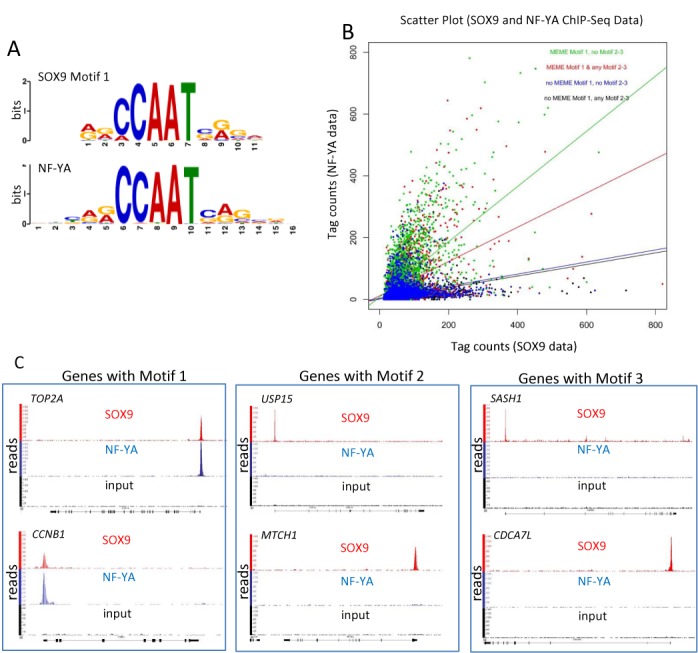
SOX9 and NF-Y co-occupy many target genes. (**A**) SOX9 and NF-YA share high homology of Motif 1 sequences identified by TOMTOM analysis. (**B**) Scatter plot of SOX9 and NF-YA peaks containing different combinations of Motif 1 and/or Motif 2–3. Different colors of dots represent genes with following Motifs: Motif1 only (green), Motif 1 with Motif 2 and/or Motif 3 (red), no Motifs 1, 2 or 3 (blue) and no Motif 1 but Motif 2 and/or Motif 3 (black). X- and y-axes represent number of tags for NF-YA and SOX9 peaks, respectively. (**C**) Representative readouts of peaks from SOX9 ChIP-seq (red), NF-YA ChIP-seq (blue) and an input control (black) for indicated genes. Peaks from SOX9 and NF-YA ChIP-seq analyses overlap only on Motif 1 genes.

TOMTOM analysis (15) further demonstrated that Motif 1 sequence contained the core DNA-binding element CCAAT, flanked by 5′ AG and 3′ CA/GG nucleotides sequence, which are identical to the preferred NF-YA binding motif obtained from the JASPAR CORE database (*E*-value of 1.7e-11) (Figure 2A). The NF-YA ChIP-seq data from ENCODE (Accession number: ENCSR000EGR; k562, human leukemia cells) contained 7647 peaks, of which 2564 overlapped with our SOX9 ChIP-seq peaks. NF-YA is a subunit of the heterotrimeric NF-Y transcription factor and therefore NF-YA ChIP-seq peaks represent binding sites for NF-Y. This was confirmed by NF-YB ChIP-seq, peaks of which were identical to those of NF-YA (26). To visualize the correlation between the SOX9 and NF-Y ChIP-seq data, we determined the number of tags in the NF-YA ChIP for the genomic regions corresponding to all of the 17 847 SOX9 peaks, regardless of whether they overlapped with a called NF-Y peak or not. Note that genomic locations that had only a NF-YA peak but no SOX9 peak were excluded from the analysis. SOX9 peaks were further annotated with MEME Motif 1 and Motif 2 and/or 3 using the FIMO software (16). The scatter plot in Figure 2B represents the SOX9 peak tag counts versus the NF-YA data tag counts for the 17 847 SOX9 peaks, which are color-coded depending on the presence or absence of Motifs 1 (NF-Y consensus) and Motifs 2/3 (SOX9 consensus). Whereas the data points broadly scatter, the plot clearly demonstrates that the SOX9 peaks with Motif 1 also have higher NF-YA peak metrics (green and red), whereas SOX9 peaks without Motif 1 (blue and black) have low NF-YA peak metrics. Linear regression lines confirm the higher correlation between SOX9 and NF-YA peak metrics with Motif 1. Among the SOX9 peaks without Motif 1 (blue and black), the presence of the SOX9-consensus (Motif 2/3; blue) has only a minimal effect on the number of SOX9 tags, whereas overlapping SOX9 and NF-Y peaks with Motif 1 were greatly enriched in the promoter region (75% within 3000 bp of TSS), leaving only 25% located outside promoter regions, including in distal enhancers. Taken together, the results of our statistical analysis of promoter sites enriched for SOX9 and NF-YA strongly suggest that both proteins likely bind to the same genomic sites.

We then investigated individual NF-Y target genes in the light of suggested cooperation between SOX9 and NF-YA. *TOP2A, CCNB1, CCNB2* and *CDK1* are all well characterized NF-Y transcriptional targets through CCAAT motifs. Alignment of SOX9 and NF-YA ChIP-seq peaks revealed that the peaks overlapped precisely in the proximal promoters of *TOP2A, CCNB1* (Figure 2C, left), *CCNB2* and *CDK1* (Supplementary Figure S2). Most other motif 1 genes also showed overlapping SOX9 and NF-YA peak sequences (data not shown). Note that Motif 2 genes (represented by *USP15* and *MTCH1*) or Motif 3 genes (represented by *SASH1* and *CDCA7L*) had SOX9 peaks, but not NF-YA peaks (Figure 2C).

To confirm that NF-YA binding sites are conserved among different cell types, we aligned NF-YA binding peaks on the representative genes, including CCNB1, CDK1 and TOP2A, using three publicly accessible NF-YA ChIP-seq data from UCSC website (HeLa-S3, Accession number: wgEncodeEH002066; GM12878, Accession number: wgEncodeEH002064; K562, Accession number: wgEncodeEH002021). A total of 72 out of randomly selected 100 SOX9 peaks in the proximal promoters had precisely overlapping peaks in all three NF-YA ChIP-seq, indicating conserved binding sites of NF-YA on those target genes. Three representative peak alignments are shown in Supplementary Figure S3. We verified randomly selected 15 peaks by NF-YA ChIP-qPCR in HT-29 cells (Supplementary Figure S4). The results suggested that the rest of NF-YA peaks obtained from other cells can also be expected in HT29 cells.

**Figure 3.**
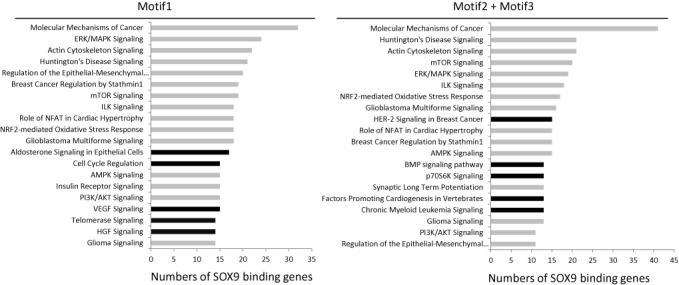
Pathway analysis of ChIP-Seq-identified SOX9 target genes. SOX9 target genes identified based on ChIP-seq were classified into two groups based on motif and pathway analysis was performed using IPA software. The top 20 pathways (*P* < 0.05) ranked by number of genes are presented. Black bars represent pathways unique to each group.

The peaks obtained by ChIP-seq and used for the motif alignment were confirmed by ChIP-qPCR with anti-SOX9 and anti-NF-YA antibodies in separate ChIP analysis of HT29 cells (Supplementary Figure S5A and S5B) and HCT116 cells (data not shown), which validated the ChIP-Seq data for regions highly enriched for SOX9 and NF-YA binding and confirmed that the findings were consistent in two colorectal cancer cell lines. These experiments confirmed that sites highly enriched in SOX9 binding overlap the NF-YA binding sites around the TSSs (within 500 bp of TSSs) of *TOP2A, CCNB1, CCNB2* and *CDK1* in colorectal cancer cells. In order to further confirm that SOX9 peaks are specific to some target sites and not to all of NF-YA binding sites, we verified by SOX9 ChIP-qPCR as well as NF-YA ChIP-qPCR in both HT-29 cells and HCT116 that only NF-YA has high binding affinity to some of its target genes, such as the promoter region of *LRIF1*, but not SOX9 (Supplementary Figure S6A and S6B). This is additional evidence that eliminates the possibility that the overlapping peaks of SOX9 and NF-YA may have simply reflected their protein to protein affinity or the affinity of anti-SOX9 antibody to NF-Y. Pathway analysis using IPA (Qiagen, Hilden, Germany) suggested that most of the SOX9 target genes related to cell cycle regulation were unique to Motif 1 genes, whereas genes involved in development and bone morphologic protein signaling were identified as having Motif 2 and/or Motif 3 (Figure 3). Although the number of genes that involved in differentiation was <10, and they, therefore, do not appear on the graph, some intestinal specific differentiation markers, such as lysozyme and defensins have Motif 3, which is the inverted repeat of the SOX9 consensus sequence. Despite similarities in the signaling pathways of Motif 1 and Motif 2 and/or 3, the genes in each group differ with regard to motif. For example, genes with Motif 1 in Molecular Mechanisms of Cancer pathway include *MAPK1, BRAF, SMAD2, FZD5, FZD8, NOTCH1* and *LRP1*, whereas genes with Motif 2 and/or Motif3 in the same pathway include *LRP5, LRP6, MDM2, CTNND1, SMAD3, SMAD4* and *SMAD7*.

### SOX9 and NF-Y bind to the target genes on CCAAT and form a complex in the nuclei of colorectal cancer cells

**Figure 4.**
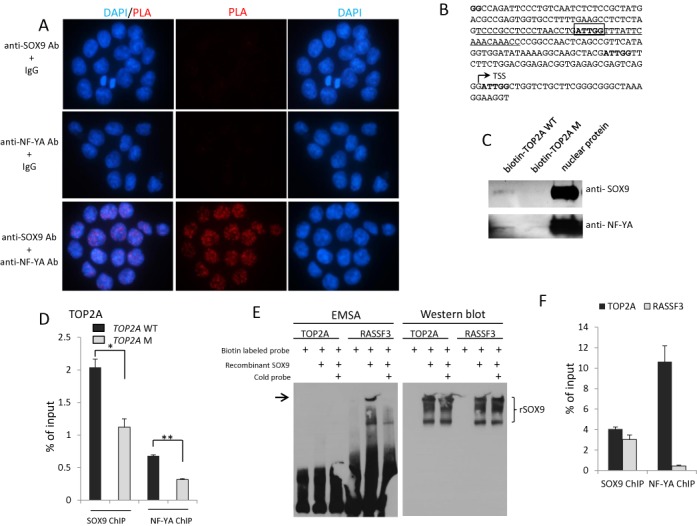
SOX9 and NF-YA likely form a protein complex. (**A**) SOX9/NF-YA interaction demonstrated by *in situ* PLA in HCT116 cells. Red signal indicates interaction of SOX9 and NF-YA. Nuclei were counterstained with DAPI. (**B**) The sequence of partial *TOP2A* promoter (−221 to + 37 bp) containing the center of SOX9 and NF-YA peaks. The center of the peak sequences of SOX9 and NF-YA enriched region, ATTGG (−106 to −102 bp), is marked by a box. Biotin labeled wild-type *TOP2A* oligo (WT *TOP2A*) and the mutated *TOP2A* (M*TOP2A*) by 1 bp were synthesized as following sequences, respectively. WT *TOP2A*: TCCCGCCTCCCTAACCTG**ATTGG**TTTATTCAAACAAACC, M*TOP2A*: TCCCGCCTCCCTAACCTG**TTTGG**TTTATTCAAACAAACC. (**C**) The biotin-mediated pull down assay showed that SOX9 and NF-YA in HCT116 nuclear extracts bind to biotin labeled wild-type *TOP2A* oligo, but 1-bp mutation of CCAAT resulted in a significant reduction of both SOX9 and NF-YA binding. (**D**) pGL3-WT TOP2A and pGL3-M TOP2A plasmids were transfected into HCT116 cells, followed by ChIP-qPCR using anti-SOX9 and anti-NF-YA antibodies 48 h post-transfection. A total of 1-bp mutation of CCAAT reduced the SOX9 and NF-YA binding to *TOP2A* promoter. (**E**) EMSA (Figure 4E, left) was performed with biotin-labeled oligonucleotides for *TOP2A* (*TOP2A* bio-F: 5′- biotin-TCCCGCCTCCCTAACCTGATTGGTTTATTCAAACAAACC) and *RASSF3* (*RASSF3* bio-F: 5′-biotin-ATGACAAGGTCTTTTTGAAGTCACCTGGAATGCTCCCTTT). Both sequences contain the SOX9 peak center obtained from our ChIP-seq. Recombinant human SOX9 protein directly binds to *RASSF3*, but not *TOP2A*. western blot (Figure 4E, right) was performed on the same membrane used for EMSA to detect recombinant human SOX9 protein. (**F**) SOX9 and NF-YA ChIP-qPCR analysis showed that SOX9 binds to both *TOP2A* and *RASSF3* (Motif 3), while NF-YA binds only to *TOP2A*, but not to *RASSF3*.

The overlapping SOX9 and NF-Y binding sites suggested a possible direct association between SOX9 and NF-Y in the chromatin. To test whether SOX9 and NF-Y interact in the nuclei of colorectal cancer cells, we performed *in situ* PLA on HT29 and HCT116 cells. *In situ* PLA results in high concentration of fluorescence only if two proteins are in close proximity (<40 nm) that indicates protein to protein interaction. *In situ* PLA using anti-SOX9 and anti-NF-YA antibodies showed positive signals in the nuclei of HCT116 (Figure 4A) and HT29 (Supplementary Figure S7) cells, indicating that SOX9 and NF-Y likely form a protein complex.

To determine whether CCAAT is necessary for SOX9 and NF-YA binding to the target genes, we performed biotin-mediated pull down assay using human wild-type *TOP2A* oligo (*TOP2A* WT) and the mutated *TOP2A* oligo (*TOP2A* M). There are four ATTGG sequences on the human TOP2A promoter (−221 to +37 bp), one of which is located in the center of SOX9 peak obtained from our ChIP-seq analysis (−106 to −102 bp) (Figure 4B). For the mutated *TOP2A* oligo, a mutation by 1 bp was made in the CCAAT to CCAAA in this location. Both SOX9 and NF-YA bind to wild-type *Topo2A* promoter, and the mutation in CCAAT resulted in a significant reduction of both SOX9 and NF-YA binding *in* *vitro* (Figure 4C). This confirmed that SOX9 and NF-Y both require CCAAT to bind to their target genes. This was further demonstrated by overexpression of wild-type and mutated TOP2A plasmids, followed by ChIP-qPCR, showing that 1-bp mutation of CCAAT reduced both SOX9 and NF-YA binding to TOP2A promoter (Figure 4D). This mutation also reduced the promoter activity of TOP2A (Supplementary Figure S8). To test whether or not SOX9 directly binds to DNA through CCAAT motif without NF-Y, we performed EMSA assay using recombinant human SOX9 proteins (Figure 4D). While recombinant SOX9 clearly bound to *RASSF3* (Motif 3 sequence), it did not bind to *TOP2A* (Motif 1) promoter sequences (Figure 4E). Prior to this experiment, we confirmed SOX9 binding to both *TOP2A* and *RASSF3* by ChIP-qPCR using HT-29 (Figure 4F). In addition, HMG DNA binding domain did not affect SOX9 binding to the promoters of *CCNB1, CDK1* or *TOP2A* (Supplementary Figure S9C). Taken together, these results strongly suggest that SOX9 requires NF-Y to bind to the target DNA through CCAAT.

**Figure 5.**
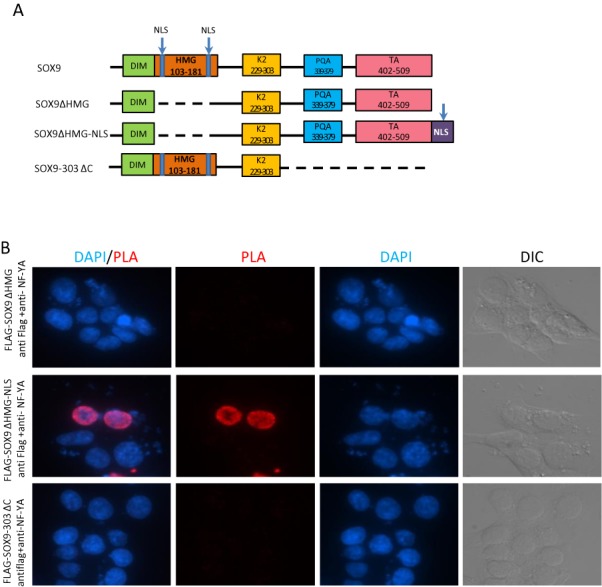
SOX9 C terminus is responsible for the interaction with NF-YA. (**A**) Schematic representation of structures of human wild-type and mutant SOX9 expressed from indicated constructs. DIM: dimerization domain; HMG domain: the DNA-binding domain; K2 and TA: transactivation domains; PQA: proline-, glutamine- and alanine-rich domain; NLS: nuclear localization sequence. (**B**) *In situ* PLA using anti-FLAG and anti-NF-YA antibodies in HCT116 cells transfected with FLAG-tagged mutant SOX9 constructs depicted in (A). Nuclei were counterstained with DAPI.

To further identify the SOX9 domain responsible for binding to NF-Y, we transfected HCT116 cells with expression constructs encoding FLAG-tagged wild-type SOX9 (FLAG-WT SOX9); C-terminus-deleted SOX9, carrying 303 amino acids from the N-terminus (FLAG-SOX9–303 ΔC); and HMG domain-deleted SOX9 fused with a nuclear localization signal (NLS) at the C-terminus (FLAG-SOX9 ΔHMG-NLS) (Figure 5A). FLAG-SOX9 ΔHMG-NLS was generated to compensate for the lack of an NLS in HMG-deleted SOX9. We first confirmed by western analysis that each FLAG-tagged protein was expressed (Supplementary Figure S9A), and, immunofluorescence analysis with anti-FLAG antibody, that the fused extra NLS enabled SOX9 to translocate into the nucleus (Supplementary Figure S9B) in HCT 116 cells. *In situ* PLA using anti-FLAG and anti NF-YA antibodies was then performed on HCT116 cells transfected with constructs encoding the three SOX9 variants. As shown in Figure 5, neither HMG domain-deleted SOX9 nor C terminus deleted SOX9 formed protein complex with NF-Y, whereas Flag-SOX9 ΔHMG-NLS did. Taken together, our data show that the C-terminus of SOX9 is required for interaction with NF-Y in the nucleus (Figure 5B).

### NF-Y recruits SOX9 to CCAAT promoters whereas SOX9 is necessary for transactivation of target genes by NF-Y

**Figure 6.**
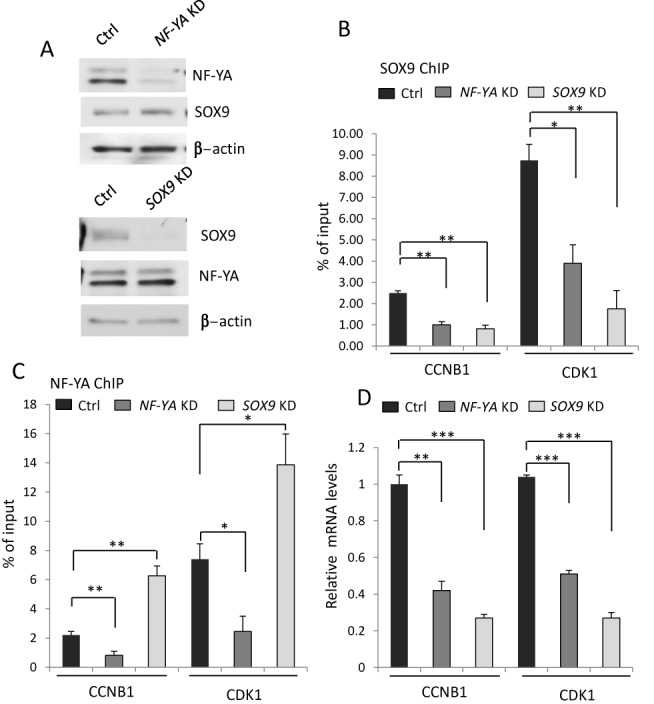
Both SOX9 and NF-Y are required for transcriptional regulation of target genes. (**A**) siRNA-mediated NF-YA and SOX9 knockdown in HCT116 cells were confirmed by western analysis. (**B**) SOX9 ChIP-qPCR analysis showed that both NF-YA knockdown cells and SOX9 knockdown cells resulted in decreased binding affinity of SOX9 to *CCNB1* and *CDK1* promoters compared to the cells treated with control siRNA (Ctrl). (**C**) NF-YA ChIP-qPCR analysis showed that decreased NF-YA expression resulted in reduction of NF-YA binding affinity to *CCNB1* and *CDK1*, however, SOX9 knockdown enhanced NF-YA binding affinity to these genes. (**D**) In NF-YA knockdown cells, expression of the corresponding mRNAs was downregulated. Upon SOX9 knockdown, although NF-YA binding affinity was enhanced, expression of the corresponding mRNAs was downregulated. *P < 0.05, **P < 0.01, ***P < 0.001.

NF-Y binding to the CCAAT motifs plays a role in the opening of target promoter chromatin and facilitates the recruitment of many cofactors to modulate transcriptional activity (27,28). To determine whether NF-Y also mediates SOX9 recruitment to the CCAAT boxes of target promoters, we carried out siRNA-mediated knockdown of *NF-YA* in HCT116 cells followed by SOX9 ChIP analysis (Figure 6A). *NF-YA* knockdown led to a significant decrease in SOX9 affinity to the promoters of *CCNB1* and *CDK1* (Figure 6B). This observation suggested that NF-Y recruits SOX9 to the target promoters. As expected, upon *NF-YA* inactivation, expression of both *CCNB1* and *CDK1* was significantly downregulated (Figure 6D). Next, to further investigate the functional aspect of SOX9 occupancy on these genes, we knocked down *SOX9* in HCT116 cells and measured NF-YA occupancy, as well as expression levels of the same target genes (Figure 6A). *SOX9* knockdown led to enhanced affinity of NF-YA binding to the *CCNB1* and *CDK1* promoters (Figure 6C). Although one might expect upregulated expression of NF-Y targets with enhanced NF-YA binding, expression of both *CCNB1* and *CDK1* was significantly downregulated in *SOX9* knockdown cells (Figure 6D). Taken together, these data support the conclusion that SOX9 is recruited by NF-Y to the promoters of some key cell cycle regulatory genes, and that SOX9 is required for the NF-Y transactivation of these genes.

## DISCUSSION

SOX9 has been shown to be critical for cell differentiation in diverse tissues. Recent ChIP-seq analyses as well as ChIP-on-chip studies in SOX9 expressing tissues have revealed many tissue specific SOX9 transcriptional targets. However, there has not been a report of genome-wide ChIP analysis in intestinal or there have been few reports identifying SOX9 targets in intestinal epithelium or colorectal cancer cells. Applying the motif discovery tool MEME to SOX9 ChIP-seq peaks in HT29 cells, we identified a *de* *novo* function of SOX9, as a cofactor of NF-Y complex through CCAAT, in addition to its function as a transcription factor through its known consensus binding sequences, ^A^/_T_^A^/_T_CAA^A^/_T_G. Furthermore, SOX9 binding through CCAAT at promoters is selective to some of the NF-Y binding sites, while SOX9 itself does not directly bind to target DNA (Supplementary Figure S6). As is the case with many other transcription factors, cofactors of SOX9 have been identified and shown to be required for precise activation and inactivation of some genes. Those cofactors include exportin 4 (Exp4) (29), steroidogenic factor 1 (SF-1) (30), p300 (31), Smad3 (32) and PGC-1α (33). In any case, however, SOX9-dependent transactivation of target genes has been shown to be mediated by recognizing a specific heptameric DNA sequence ^A^/_T_^A^/_T_CAA^A^/_T_G through its HMG-box domain. Our data obtained using human colorectal cancer cells show that some cell cycle regulatory genes exhibit enriched SOX9 binding to the CCAAT motif, and that those SOX9 enriched sites overlap precisely with NF-Y binding peaks in the proximal promoters. Those genes lack the traditional binding sequence ^A^/_T_^A^/_T_CAA^A^/_T_G within the peak sequences. Further, our mutagenesis analyses as well as *in vitro* binding assays confirmed CCAAT as the interaction sequences of SOX9 and NF-Y complexes. Our data showed that SOX9 does not require its DNA binding domain and that SOX9 binding on the target genes are NF-Y dependent. This indicates that SOX9 binding is through NF-Ys on CCAAT and that SOX9 does not directly bind to CCAAT.

There have been four SOX9 ChIP data reported (21–23,34), but none of them indicated SOX9 association with NF-Y on CCAAT. The potential reason includes that two reports were based on SOX9 ChIP-on-chip with limited genes placed on the chips (21,22). Two SOX9 ChIP-seq analyses were also performed on less proliferative tissues, hair follicle stem cell niches (23) and chondrocytes (34). In the normal physiology of less proliferative tissue, the affinity of SOX9 binding to CCAAT may be significantly lower than those to the consensus sequences ^A^/_T_^A^/_T_CAA^A^/_T_G and, therefore, may not be significant. However, it could be the case that this might be a unique feature of SOX9, either tissue specific or context specific. In order to determine whether SOX9 interaction with NF-Y could be limited to cancer cells, and not in normal intestinal epithelium, SOX9 ChIP-seq on normal intestinal epithelial cells should be performed.

New finding of SOX9 affinity to CCAAT could also be associated with the previously reported differential roles of SOX9 expressed at high and low levels (6). At a high expression level, SOX9 may bind through all three motifs, Motif 1, 2 and 3, which occur in differentiation marker genes, as well as other genes that lead to reduced proliferation, whereas at a low expression level, SOX9 may bind predominantly to CCAAT motif genes that have relatively higher affinity for SOX9. In fact, in our ChIP-seq assays, SOX9 enrichment was observed on some Paneth cell differentiation markers, such as lysozyme and defensins, suggesting that SOX9 plays a role in Paneth cell differentiation through the classical motif, Motif 2 and/or 3. This is consistent with previous studies in which SOX9 is shown to play a crucial role as a transactivator in differentiation, such as in the outer root sheath of the hair follicle and in chondrocytes (21,23). Based on our ChIP-seq analysis, as in other tissues, SOX9 is likely involved in cell differentiation through its consensus binding sequences.

NF-Y, a ubiquitously expressed heterotrimeric complex in which NF-YA is the DNA-binding subunit, has been shown to be a key regulator in cell cycle progression, particularly at G2/M phase (10,11,35–40). NF-YA has been shown to be critical for development and cell proliferation in embryo (25), as well as in adult tissues such as liver (41) and hematopoietic stem cells (35). As activation of cell cycle regulatory genes by NF-Y has been demonstrated in various studies, in which NF-Y binding to DNA was inhibited either by the expression of the dominant negative NF-Y subunit or by inactivation of the *NF-Y* gene (36,42). Inhibition of DNA binding by NF-Y leads to growth arrest at multiple phases of the cell cycle and the *in vivo* significance of NF-Y was evidenced by NF-YA knockout in mice, which resulted in early embryonic lethality (25). Recent studies demonstrated the role of NF-Y in stem cell progression and maintenance, whereas expression is downregulated during differentiation. However, NF-Y is still active in differentiated neuronal cells and conditional deletion of *NF-YA* in mouse brain induces progressive neurodegeneration (43), indicating the crucial role of NF-Y also in postmitotic differentiated cells. The roles of NF-Y as an essential regulator of the pluripotent state in human embryonic stem cells (ESCs) and cell specification was recently demonstrated, whereby NF-Y promotes chromatin accessibility of cell type-specific transcription factors (26). In light of this report, an interesting future direction is to reveal whether or not SOX9 may also play a role by binding with NF-Y to its non-traditional, cell type-specific enhancers in colorectal cancer cells. And this can be done only by performing NF-Y ChIP-seq using colorectal cancer cells. SOX9 is established as being expressed in the proliferative compartment in the intestinal epithelium, where terminally differentiated Paneth cells and quiescent stem cells reside, which express high expression of SOX9. Whether SOX9 may be involved in NF-Y target gene regulation in normal intestinal mucosal cells or whether the interaction of SOX9 and NF-Y is entirely specific to the context of cancer should be also addressed in a future study.

Our *in* *situ* PLA results indicated that SOX9 and NF-Y comprise a protein complex in the nucleus. ChIP analysis of NF-Y knockdown cells strongly suggest that NF-Y is necessary for SOX9 binding to the CCAAT sites. This observation is consistent with the roles of NF-Y, in that histone-fold domain protein NF-Y pre-sets the promoter architecture that allows other regulatory proteins access (11). On the other hand, SOX9 knockdown cells showed decreased expression of NF-Y target genes, indicating that SOX9 is critical for NF-Y mediated transactivation of these genes. Taken together, the data show that SOX9 binds to some cell cycle regulatory genes as a cofactor of NF-Y through the CCAAT motif and facilitates NF-Y mediated transactivation. In SOX9 depleted cells, although NF-Y target cell cycle genes exhibited downregulation, the cells did not show significant cell cycle arrest. This is perhaps due to the presence of many other SOX9 target genes that regulate proliferation rate directly or indirectly, the expression of which is also altered upon SOX9 inactivation. For example, insulin-like growth factor (IGF)-binding protein 4 (IGFBP-4), an inhibitor of the IGF/IGF receptor pathway, is a direct transcriptional target of SOX9, whose expression is significantly downregulated upon inactivation of SOX9 (19).

SOX9 binds to an inverted repeat of a heptameric sequence, and this dimeric binding is necessary for SOX9-dependent transactivation of downstream genes, at least in some tissues including cartilages (44). For example, SOX9 dimerization mutants have been identified in some campomelic dysplasia patients, suggesting the importance of the repetition of the sequence (24). Our ChIP-seq data suggested that some of the genes with high affinity for SOX9 contained inverted repeats of putative sequences (Motif 3); however, such repeats were also observed in many of the peak sequences of Motif 2 genes, allowing spacers of 2–14 bp. We hypothesize that dimerization of SOX9 is likely also required for transactivation of some genes in colorectal cancer cells. However, functional assays of each gene are needed to confirm the mechanism in detail.

SOX9 has also been shown to be indispensable for stem cell maintenance in some tissues, such as hair follicle, biliary and pancreatic ductal epithelium and mammary epithelium (23,29,44). Our ChIP-seq analysis showed enriched SOX9 binding to some intestinal stem cell (ISC) marker genes such as *BMI1, Lrig1* and *Lgr5*, also suggesting a potential role of SOX9 for ISC regulation. SOX9 consensus sequences are identifiable within the peak sequences of each of these genes. A recent study demonstrated the significance of cell type specific NF-Y regulation of master ESC transcription factor expression through distal enhancer binding (26,30). Our ChIP-seq data showed enrichment of SOX9 binding to promoters of some of the major ISC markers, including *BMI1, Lrig1* and *Lgr5*. Neither CCAAT boxes nor peaks overlapping with NF-Y ChIP-seq peaks were identified, but SOX9 consensus sequences were observed within those peaks. We speculate that SOX9 may also be involved in stem cell maintenance in the intestinal epithelium. However, SOX9 is not likely to be involved in the NF-Y-mediated stem cell regulation complexes in the distal enhancer (26).

Although, SOX9 has been reported to inhibit or sustain cell proliferation in different cell types, on the other hand, SOX9 is expressed in most human colorectal cancers as well as in human colorectal cancer cell lines. Our finding of activation of proliferative genes through NF-Y, therefore, gives an insight into the oncogenic aspect of SOX9 and helps to explain why anti-oncogenic protein, SOX9 is highly expressed in some cancers including most colorectal cancer cells.

## SUPPLEMENTARY DATA

Supplementary Data are available at NAR Online.

SUPPLEMENTARY DATA
