# Supplementary material for: Context-specific role of SOX9 in NF-Y mediated gene regulation in colorectal cancer cells
Source: Nucleic Acids Res. 2015 Jun 3;43(13):6257–69. doi: 10.1093/nar/gkv568 (PMC4513854; doi:10.1093/nar/gkv568)
Supplement: SUPPLEMENTARY DATA [file supp_gkv568_nar-03416-x-2014-File008.pptx]

## Slide 1
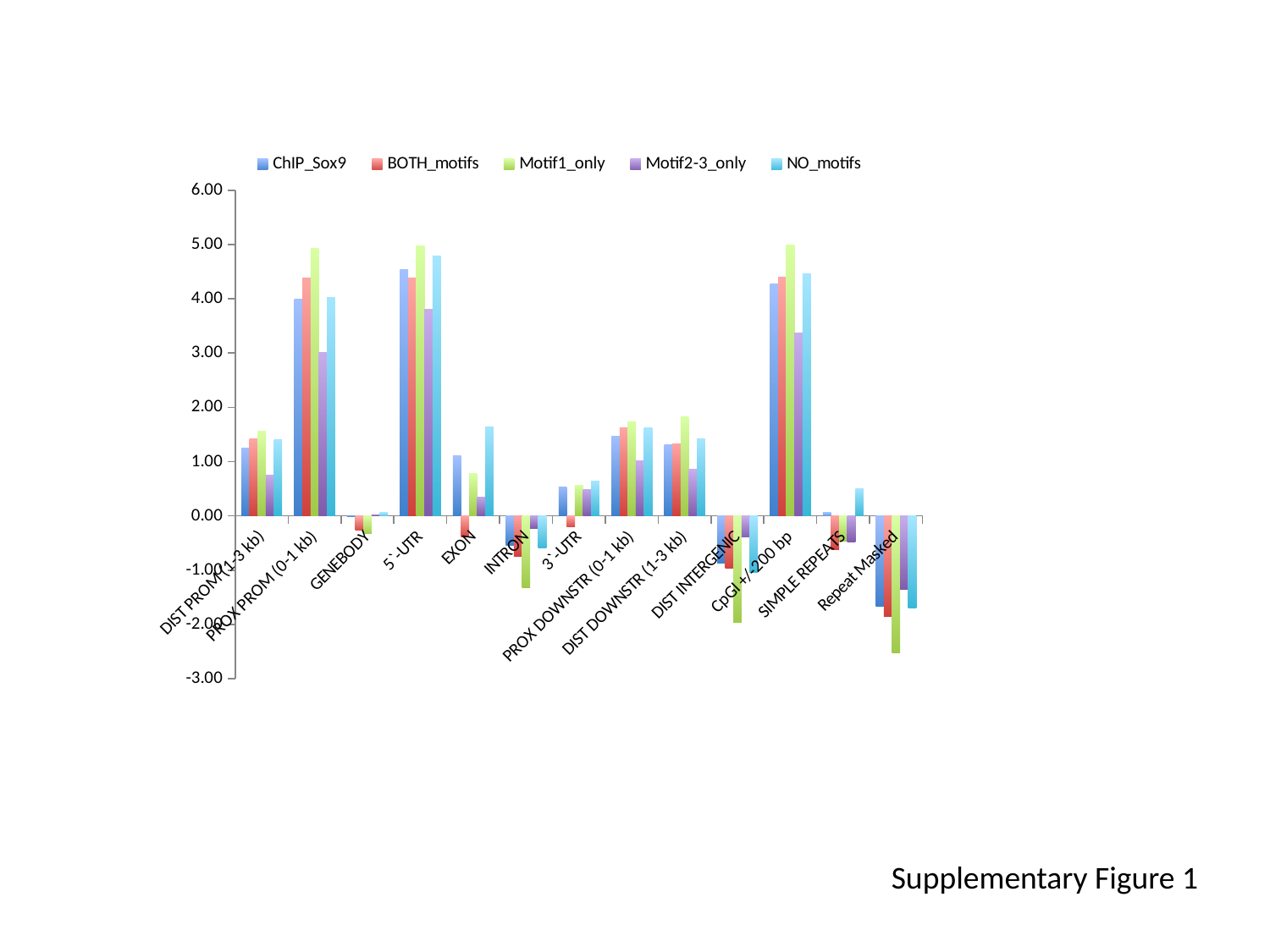

### Chart
| Category | ChIP_Sox9 | BOTH_motifs | Motif1_only | Motif2-3_only | NO_motifs |
|---|---|---|---|---|---|
| DIST PROM (1-3 kb) | 1.251940575950209 | 1.4294465920991506 | 1.5678093232122745 | 0.751050218553162 | 1.4079028357801509 |
| PROX PROM (0-1 kb) | 3.9876777706052415 | 4.382068316805644 | 4.931847274964459 | 3.0134974028838264 | 4.027920272332041 |
| GENEBODY | -0.019046941785263408 | -0.2676805236004431 | -0.32316738348204366 | 0.022204197377838807 | 0.06375994828954139 |
| 5`-UTR | 4.538647307808616 | 4.3782410517390655 | 4.980440417326321 | 3.7990417641405867 | 4.793634706530509 |
| EXON | 1.1101829177504232 | -0.3819213924650771 | 0.7764790918845923 | 0.3399982275610132 | 1.6443633763065388 |
| INTRON | -0.543985356496228 | -0.7376668501389102 | -1.324827781605497 | -0.2271798891171233 | -0.5902939220819606 |
| 3`-UTR | 0.5385269277308715 | -0.19254402835659223 | 0.5673070795028022 | 0.4871473562802005 | 0.6500287872426354 |
| PROX DOWNSTR (0-1 kb) | 1.4728815738511283 | 1.6311347603603694 | 1.7389091716400706 | 1.018700543450948 | 1.6265768928115698 |
| DIST DOWNSTR (1-3 kb) | 1.319431362148464 | 1.326491679898891 | 1.8233083819244915 | 0.8575266798470222 | 1.4175127058979062 |
| DIST INTERGENIC | -0.8699727361437188 | -0.9591843683553358 | -1.9543434794190844 | -0.38993252505759873 | -1.045546913984643 |
| CpGI +/-200 bp | 4.277002224005846 | 4.404379062329854 | 4.987238356558338 | 3.364651409323036 | 4.453307760002986 |
| SIMPLE REPEATS | 0.06160224966844097 | -0.6238099894273225 | -0.4654095050776529 | -0.473047070597358 | 0.5082694433668901 |
| Repeat Masked | -1.660323107218345 | -1.8503285343258524 | -2.5136129158807647 | -1.3532184539376166 | -1.6876666235030435 |Supplementary Figure 1
