# Supplementary figures and images for: Context-specific role of SOX9 in NF-Y mediated gene regulation in colorectal cancer cells
Source: Nucleic Acids Res. 2015 Jun 3;43(13):6257–69. doi: 10.1093/nar/gkv568 (PMC4513854; doi:10.1093/nar/gkv568)

## Slide 1
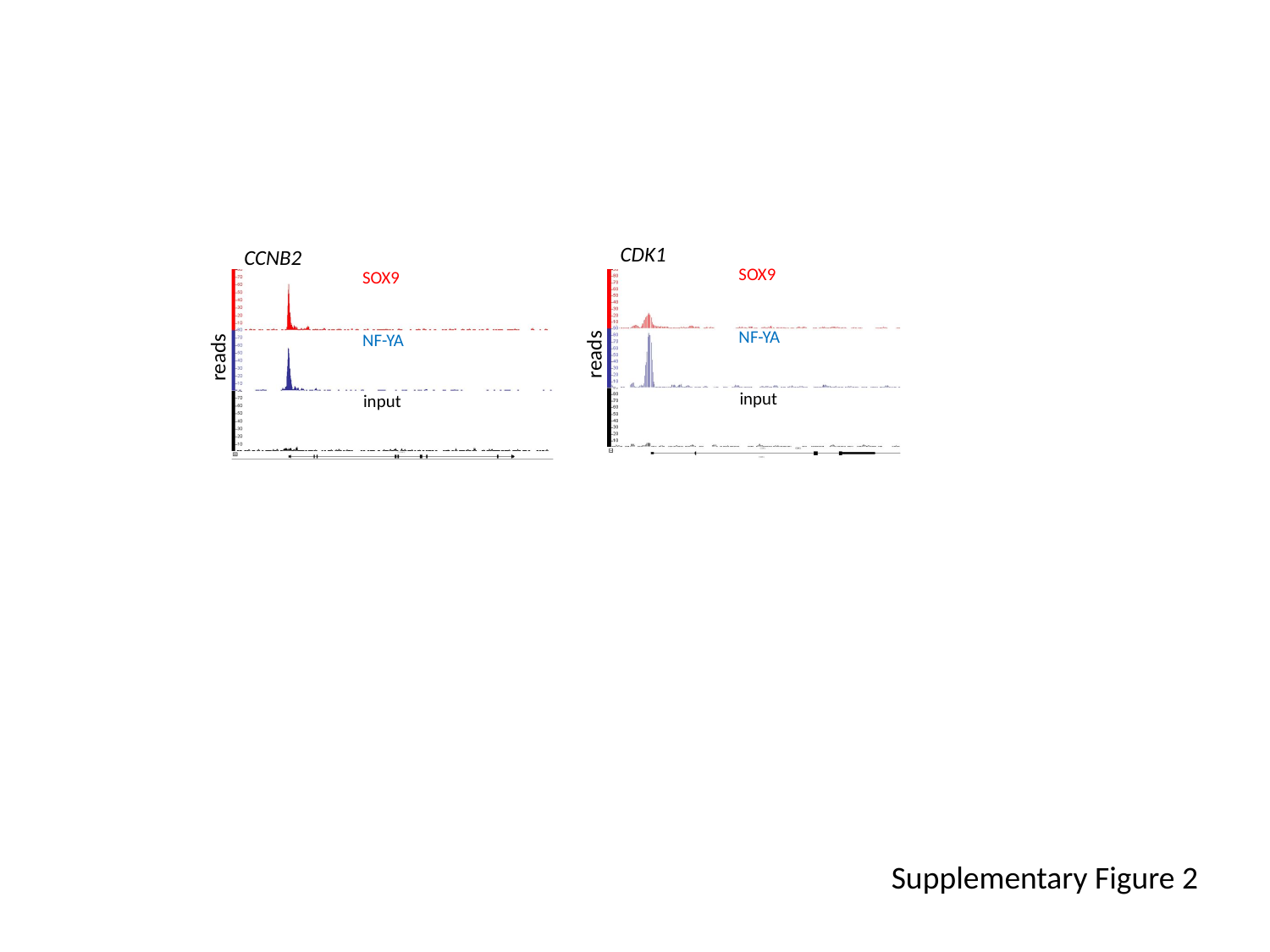

CDK1
SOX9
reads
NF-YA
input
CCNB2
SOX9
reads
NF-YA
input
Supplementary Figure 2

Supplement: SUPPLEMENTARY DATA [file supp_gkv568_nar-03416-x-2014-File009.pptx]

## Slide 1
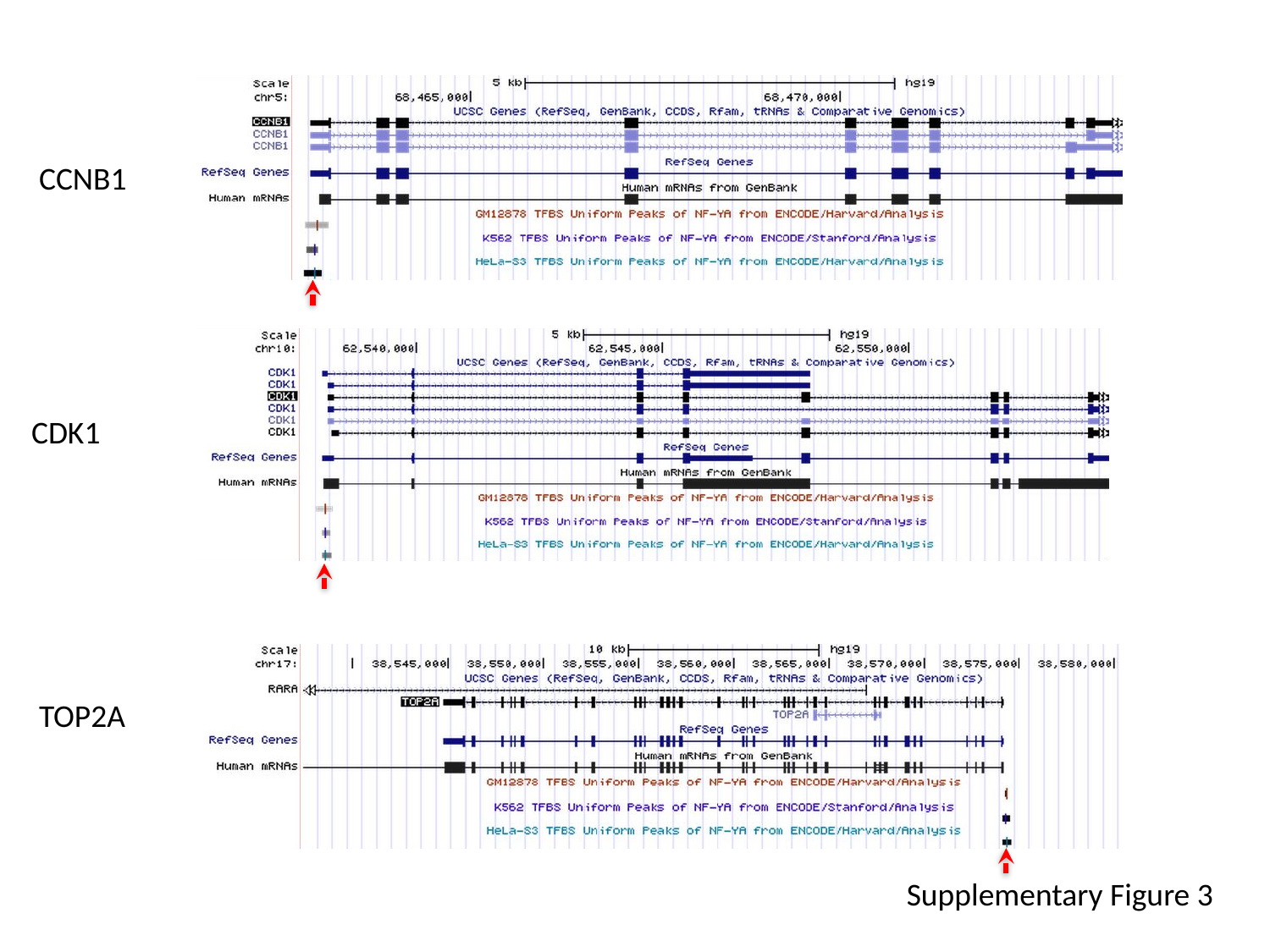

CCNB1
CDK1
TOP2A
Supplementary Figure 3

Supplement: SUPPLEMENTARY DATA [file supp_gkv568_nar-03416-x-2014-File010.pptx]

## Slide 1
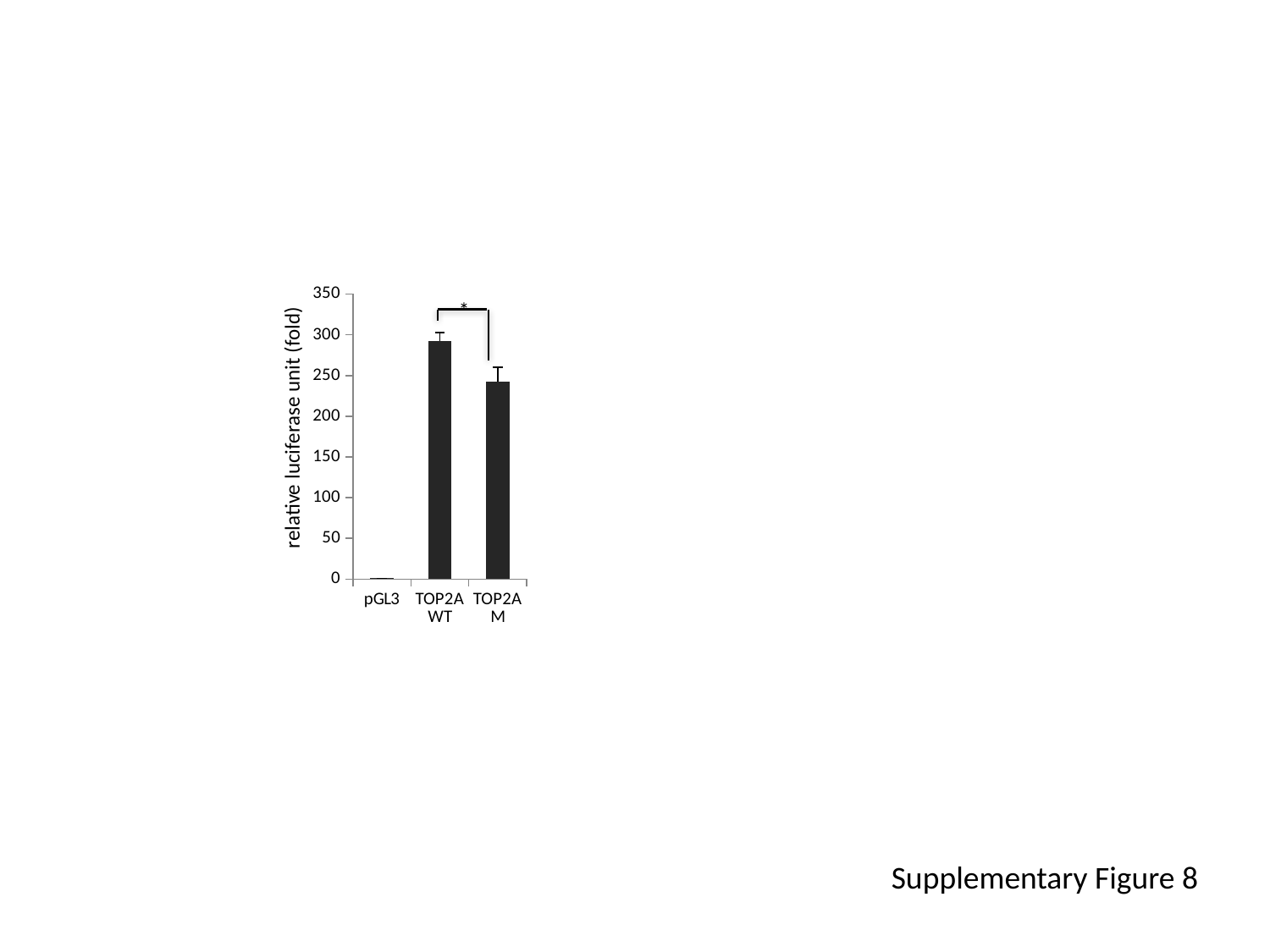

### Chart
| Category | |
|---|---|
| pGL3 | 1.0 |
| TOP2A WT | 292.5358485430948 |
| TOP2A M | 242.24552212148532 |*
relative luciferase unit (fold)
Supplementary Figure 8

Supplement: SUPPLEMENTARY DATA [file supp_gkv568_nar-03416-x-2014-File015.pptx]
