# Supplementary material for: Context-specific role of SOX9 in NF-Y mediated gene regulation in colorectal cancer cells
Source: Nucleic Acids Res. 2015 Jun 3;43(13):6257–69. doi: 10.1093/nar/gkv568 (PMC4513854; doi:10.1093/nar/gkv568)
Supplement: SUPPLEMENTARY DATA [file supp_gkv568_nar-03416-x-2014-File011.pptx]

## Slide 1
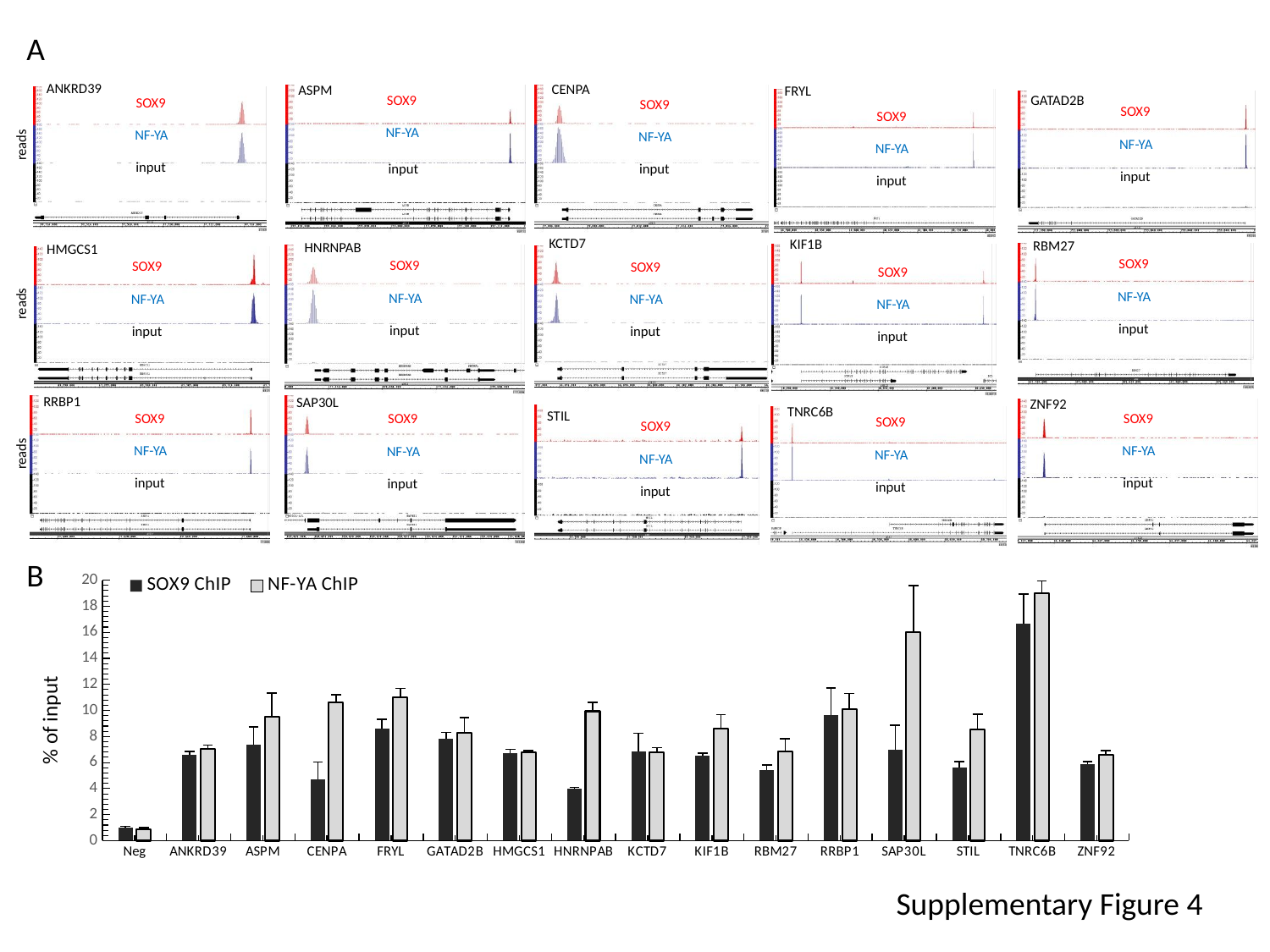

A
ANKRD39
SOX9
NF-YA
input
CENPA
SOX9
NF-YA
input
ASPM
FRYL
SOX9
NF-YA
input
SOX9
NF-YA
input
GATAD2B
SOX9
NF-YA
input
reads
KCTD7
SOX9
NF-YA
input
KIF1B
SOX9
NF-YA
input
RBM27
SOX9
NF-YA
input
HNRNPAB
SOX9
NF-YA
input
HMGCS1
SOX9
NF-YA
input
reads
RRBP1
SOX9
NF-YA
input
SAP30L
SOX9
NF-YA
input
 ZNF92
SOX9
NF-YA
input
TNRC6B
SOX9
NF-YA
input
STIL
SOX9
NF-YA
input
reads
B
### Chart
| Category | SOX9 ChIP | NF-YA ChIP |
|---|---|---|
| Neg | 0.9736658732096259 | 0.858714421590129 |
| ANKRD39 | 6.605374878628037 | 7.020561300665985 |
| ASPM | 7.4053842721527205 | 9.490981144400026 |
| CENPA | 4.717229640045931 | 10.594312940681746 |
| FRYL | 8.60088167662768 | 11.025049603283106 |
| GATAD2B | 7.846346802873602 | 8.283133382900509 |
| HMGCS1 | 6.74983527635829 | 6.801918028390741 |
| HNRNPAB | 3.962581638776533 | 9.939276708164948 |
| KCTD7 | 6.8376493617311995 | 6.784378067324573 |
| KIF1B | 6.525571600545677 | 8.632847948701732 |
| RBM27 | 5.421270741365101 | 6.883599067760237 |
| RRBP1 | 9.627265539703496 | 10.124353767499565 |
| SAP30L | 6.990275664302518 | 16.013268538269376 |
| STIL | 5.640283164305733 | 8.52328564754668 |
| TNRC6B | 16.662695313293835 | 18.98128472115027 |
| ZNF92 | 5.868376157867647 | 6.566984007075176 |% of input
Supplementary Figure 4
