# Supplementary material for: Context-specific role of SOX9 in NF-Y mediated gene regulation in colorectal cancer cells
Source: Nucleic Acids Res. 2015 Jun 3;43(13):6257–69. doi: 10.1093/nar/gkv568 (PMC4513854; doi:10.1093/nar/gkv568)
Supplement: SUPPLEMENTARY DATA [file supp_gkv568_nar-03416-x-2014-File012.pptx]

## Slide 1
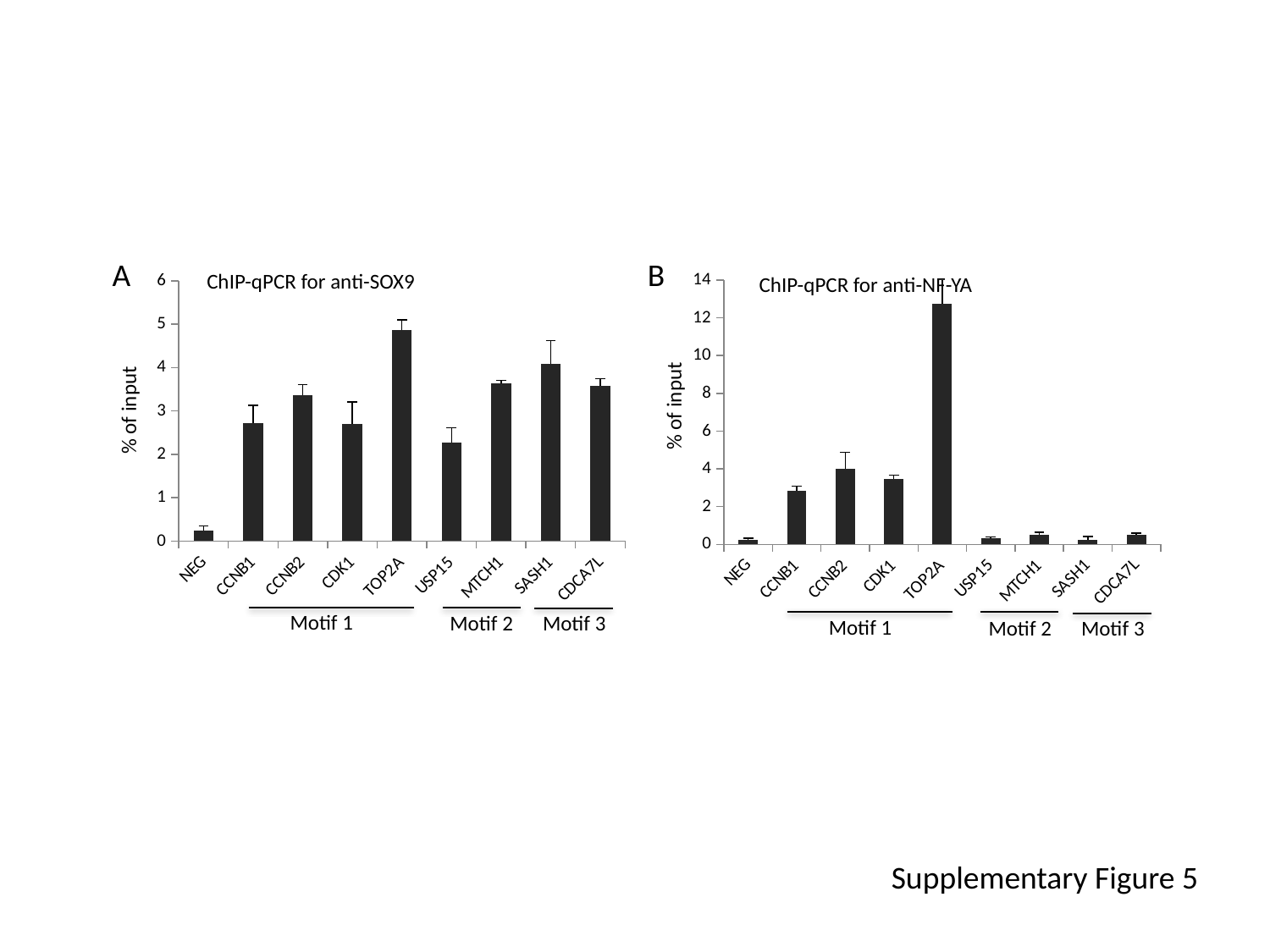

A
B
ChIP-qPCR for anti-SOX9
ChIP-qPCR for anti-NF-YA
% of input
% of input
Motif 1
Motif 2
Motif 3
Motif 1
Motif 2
Motif 3
### Chart
| Category | |
|---|---|
| NEG | 0.25 |
| CCNB1 | 2.8416091753022115 |
| CCNB2 | 4.01226366225 |
| CDK1 | 3.475216680859225 |
| TOP2A | 12.7546312263662 |
| USP15 | 0.34079536404898053 |
| MTCH1 | 0.5161663417857455 |
| SASH1 | 0.259161393196965 |
| CDCA7L | 0.519250237128066 |
### Chart
| Category | |
|---|---|
| NEG | 0.25 |
| CCNB1 | 2.72777982997018 |
| CCNB2 | 3.36795510675 |
| CDK1 | 2.690836642943448 |
| TOP2A | 4.86024929551067 |
| USP15 | 2.276324986261906 |
| MTCH1 | 3.63150013260349 |
| SASH1 | 4.08021982609233 |
| CDCA7L | 3.57232914563221 |Supplementary Figure 5
