# Supplementary material for: Context-specific role of SOX9 in NF-Y mediated gene regulation in colorectal cancer cells
Source: Nucleic Acids Res. 2015 Jun 3;43(13):6257–69. doi: 10.1093/nar/gkv568 (PMC4513854; doi:10.1093/nar/gkv568)
Supplement: SUPPLEMENTARY DATA [file supp_gkv568_nar-03416-x-2014-File013.pptx]

## Slide 1
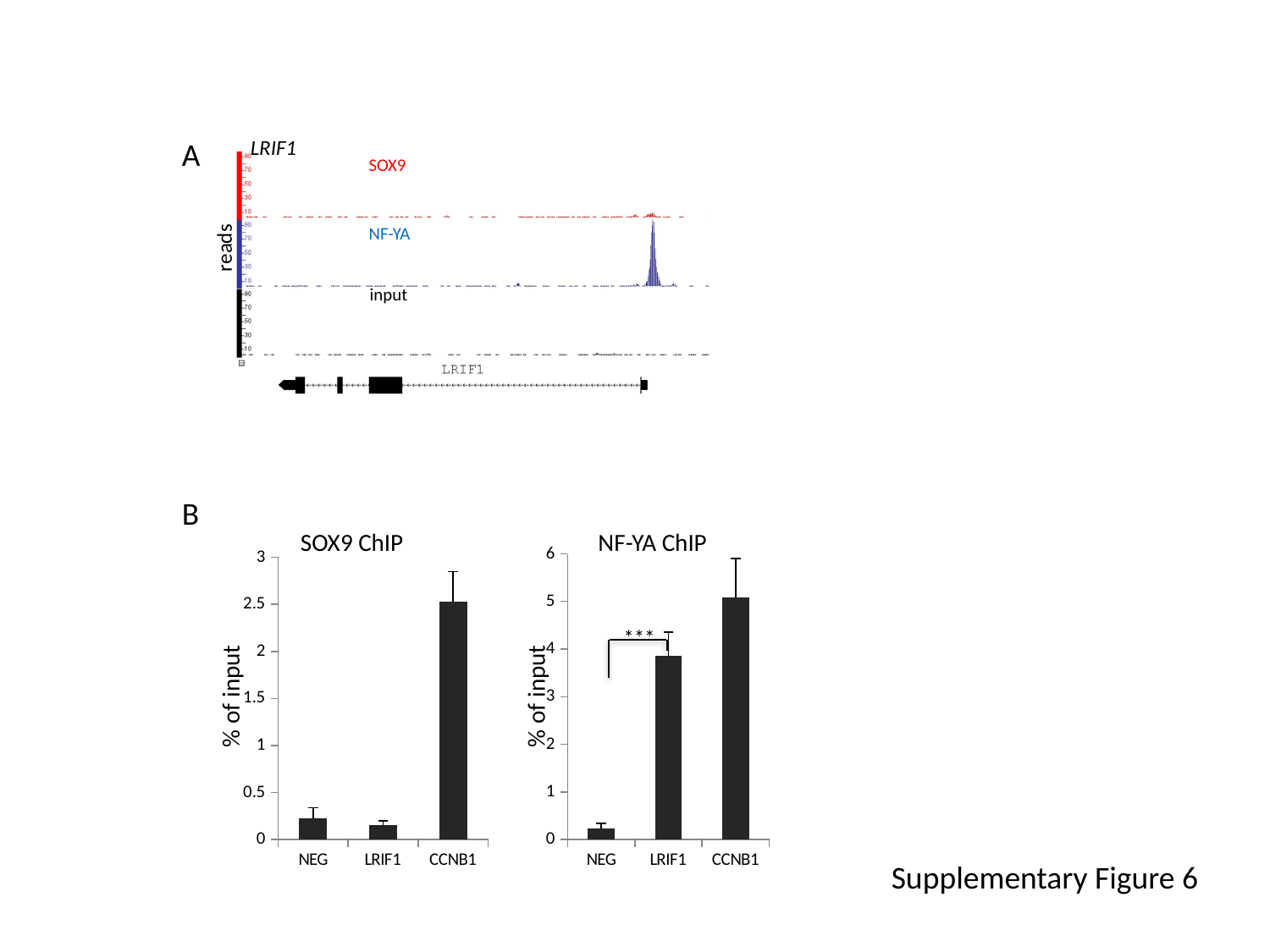

A
LRIF1
SOX9
reads
NF-YA
input
B
NF-YA ChIP
SOX9 ChIP
### Chart
| Category | |
|---|---|
| NEG | 0.23 |
| LRIF1 | 3.85 |
| CCNB1 | 5.08 |
### Chart
| Category | |
|---|---|
| NEG | 0.23 |
| LRIF1 | 0.15 |
| CCNB1 | 2.53 |***
% of input
% of input
Supplementary Figure 6
