# Supplementary material for: Context-specific role of SOX9 in NF-Y mediated gene regulation in colorectal cancer cells
Source: Nucleic Acids Res. 2015 Jun 3;43(13):6257–69. doi: 10.1093/nar/gkv568 (PMC4513854; doi:10.1093/nar/gkv568)
Supplement: SUPPLEMENTARY DATA [file supp_gkv568_nar-03416-x-2014-File014.pptx]

## Slide 1
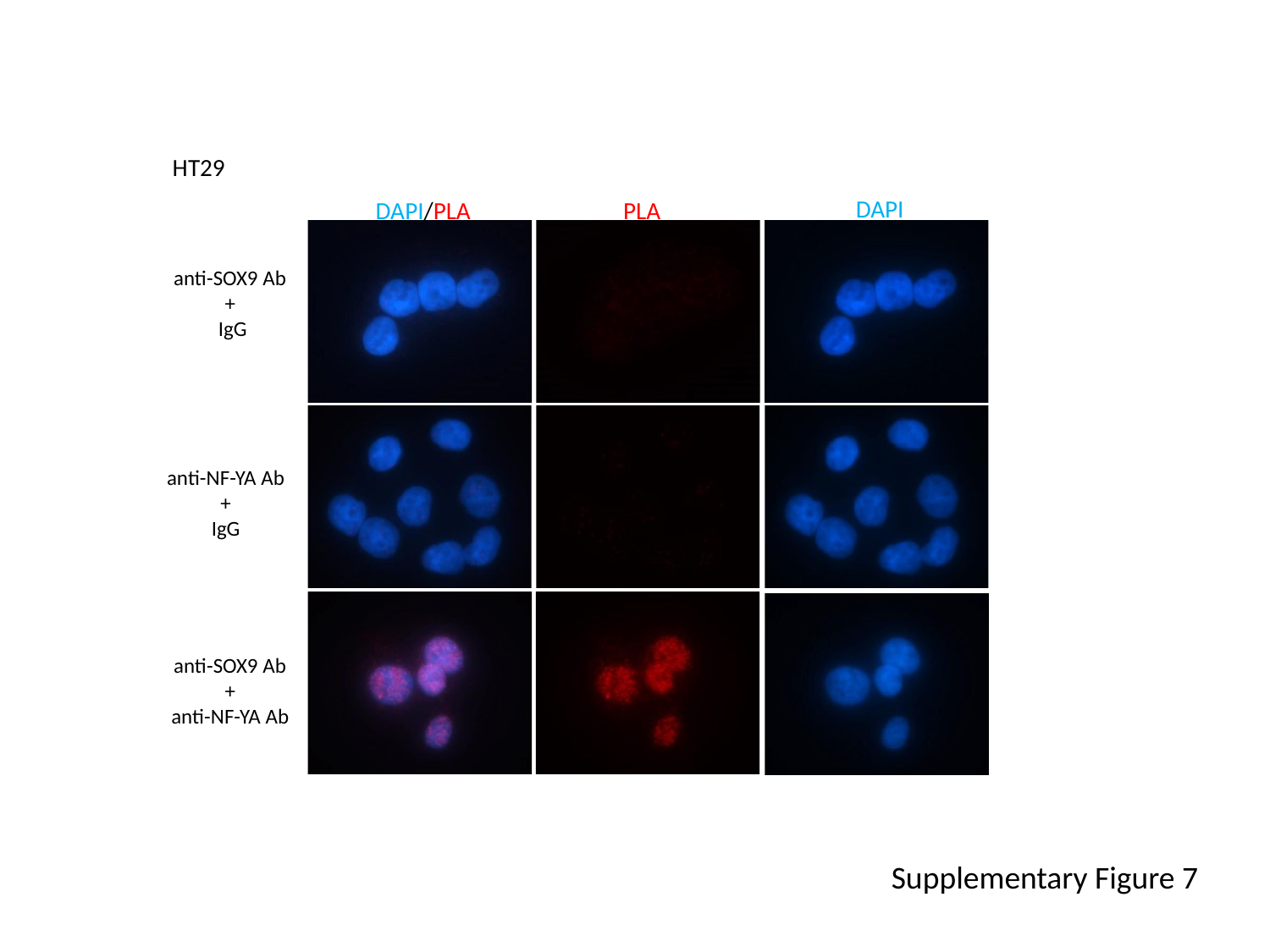

HT29
DAPI
DAPI/PLA
PLA
anti-SOX9 Ab
+
 IgG
anti-NF-YA Ab
+
IgG
anti-SOX9 Ab
+
anti-NF-YA Ab
Supplementary Figure 7
