# Supplementary material for: Context-specific role of SOX9 in NF-Y mediated gene regulation in colorectal cancer cells
Source: Nucleic Acids Res. 2015 Jun 3;43(13):6257–69. doi: 10.1093/nar/gkv568 (PMC4513854; doi:10.1093/nar/gkv568)
Supplement: SUPPLEMENTARY DATA [file supp_gkv568_nar-03416-x-2014-File016.pptx]

## Slide 1
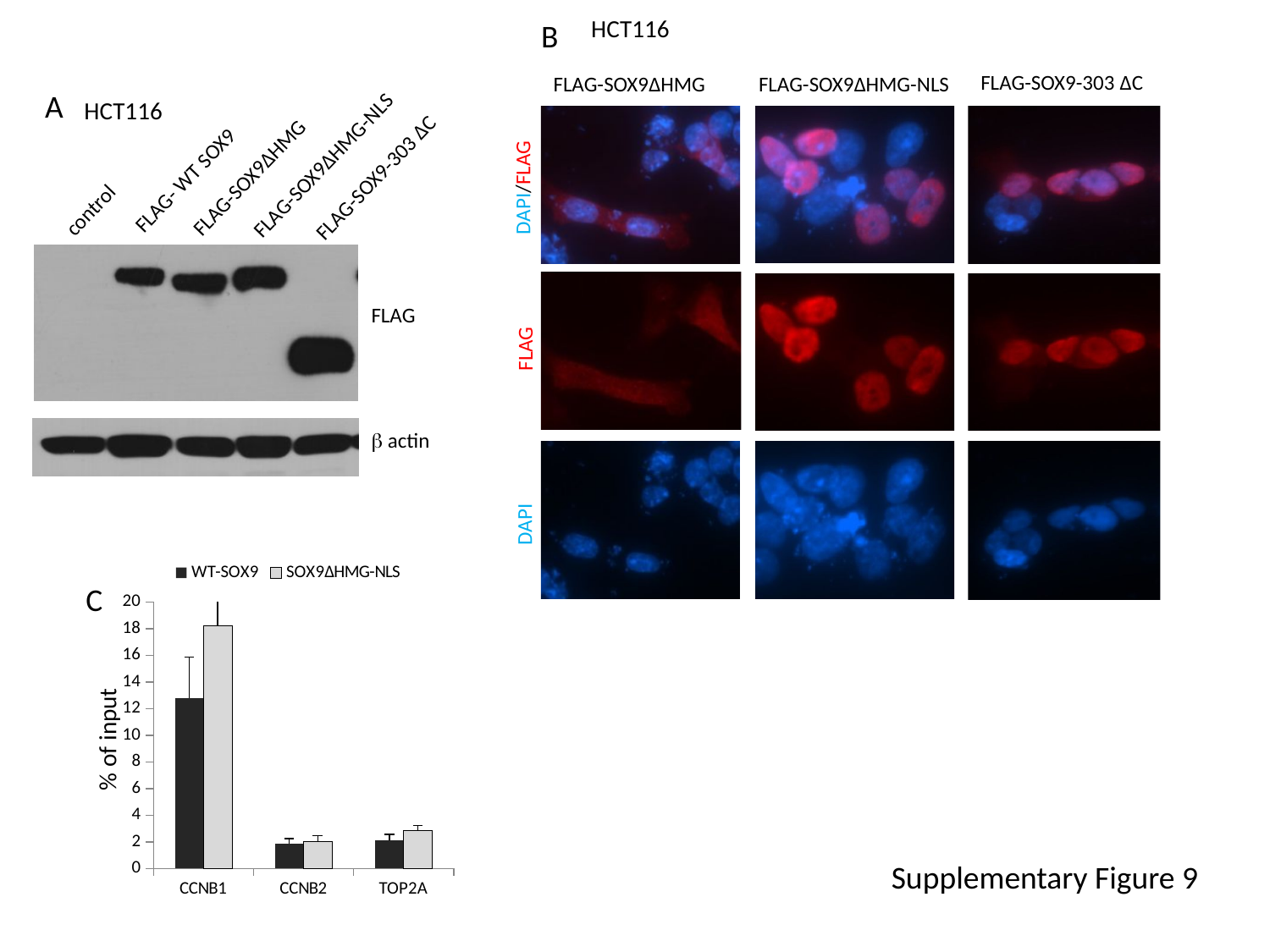

FLAG- WT SOX9
FLAG-SOX9ΔHMG-NLS
FLAG-SOX9ΔHMG
FLAG-SOX9-303 ΔC
control
FLAG
b actin
HCT116
B
FLAG-SOX9-303 ΔC
FLAG-SOX9ΔHMG-NLS
FLAG-SOX9ΔHMG
A
HCT116
DAPI/FLAG
FLAG
DAPI
### Chart
| Category | WT-SOX9 | SOX9ΔHMG-NLS |
|---|---|---|
| CCNB1 | 12.813530133280265 | 18.229792797184 |
| CCNB2 | 1.8798903136580614 | 1.9938804451664758 |
| TOP2A | 2.1402883319136934 | 2.82633038240427 |C
% of input
Supplementary Figure 9
