# Supplementary material for: Context-specific role of SOX9 in NF-Y mediated gene regulation in colorectal cancer cells
Source: Nucleic Acids Res. 2015 Jun 3;43(13):6257–69. doi: 10.1093/nar/gkv568 (PMC4513854; doi:10.1093/nar/gkv568)
Supplement: SUPPLEMENTARY DATA [file supp_gkv568_nar-03416-x-2014-File017.docx]

Supplementary Table 1.

Primers for ChIP-qPCR

| Gene | Primer Sequence |
| --- | --- |
| Negative control | Forward: 5’-TGAGCATTCCAGTGATTTATTG-3’  Reverse: 5’-AAGCAGGTAAAGGTCCATATTTC -3’ |
| CCNB1 | Forward: 5’-GATCGCCCTGGAAACGCATT-3’  Reverse: 5’-GGCCTGTTCGTGGCACTCAC -3’ |
| CCNB2 | Forward: 5’-AATGAGAACAGCGACCCGTG-3’  Reverse: 5’-CCAGGATTCAAATACCGCGTC -3’ |
| CDK1 | Forward: 5’-GCCCTTTCCTCTTTCTTTCG-3’  Reverse: 5’- TTCAAAGCAGCCAATCAGAGC-3’ |
| TOP2A | Forward: 5’-CCACCTATGAACGGCTGAGT-3’ |
|  | Reverse: 5’-CCTGTCAATCTCTCCGCTATG-3’ |
| USP15 | Forward: 5’-GCCAAGGCAAGATGCTGGAC -3’  Reverse: 5’- CGGTCGAAACCAAAAGACACC-3’ |
| MTCH1 | Forward: 5’-TGGGACACGCCGGATGTGG -3’  Reverse: 5’- GCTGCCCGCCACCATTGAC-3’ |
| SASH1 | Forward: 5’-GCGTGCCTTAGTTAGTTGGTTC -3’  Reverse: 5’- GCAACAGGGTAGCAGGAGGC -3’ |
| CDCA7L  LRIF1  RASSF3  ANKRD39 | Forward: 5’- TCCGATCACGTTCCAACTTC -3’  Reverse: 5’- GCCCTAACACTGGTAATTGCTC -3’  Forward: 5’- AGGTGACTGGCTCGCTAAAGC-3’  Reverse: 5’- GTCGGGAGAGCTTTCTTTTGC-3’  Forward: 5’- TTGAAGTCACCTGGAATGCTC-3’  Reverse: 5’- GAAACCACAAACTACACCTGG-3’  Forward: 5’- CCTTCTCGCCAAAGCTGACC-3’  Reverse: 5’- AAGCCCCGCCCTACCGTTG-3’ |
| ASPM  CENPA  FRYL | Forward: 5’- AGAGGAGCCAAACAAGTATGG-3’  Reverse: 5’- ATTGGTCCCGTGACGAGGAG-3’  Forward: 5’- CAGGGGGCAGGAGAATTTCC-3’  Reverse: 5’- AGGACAGGGGAGACTGATGG-3’  Forward: 5’- CGCTCAAAGTTGCTCCTACC-3’  Reverse: 5’- GGGCAGAGAATGATTAGAGG-3’ |
| GATAD2B  HMGCS1  HNRNPAB  KCTD7  KIF1B  RBM27  RRBP1  SAP30L  STIL  TNRC6B  ZNF92 | Forward: 5’- ACACGCCACGACGCCCATTG-3’  Reverse: 5’- GGTGAAGTCCCGGCATCTAG-3’  Forward: 5’- GAGGAAGTGGTGTGAGAGAC-3’  Reverse: 5’- ATCTCGCAGCTCCGTCATTG-3’  Forward: 5’- AATGGGACGCTGAAGCTAGG-3’  Reverse: 5’- CGTGGCGCCCTTTATAATGC-3’  Forward: 5’- GACCAATCAGTGCACGGCAGG-3’  Reverse: 5’- CCTGAGTGGTCCCTGGGTTGG-3’  Forward: 5’- CGCTCGATGTTCCTGCAGC-3’  Reverse: 5’- CAGCCAATCGTAGGGTGAGC-3’  Forward: 5’- AAGCTCTCCACCTACCTTGC-3’  Reverse: 5’- CCTGAGAACCAGTAAGAGAG-3’  Forward: 5’- GGCCAGCCATTCATCGAGC-3’  Reverse: 5’- GTCCTGACTGGCCAGCGAC-3’  Forward: 5’- AGAGTCCAGGAAGTAGCCTG-3’  Reverse: 5’- TTGGTCTCATTTGCTCTCTGG-3’  Forward: 5’- CAAGCTCGCGAAACTGAAGG-3’  Reverse: 5’- CACCAATACGTAACGGCGACC-3’  Forward: 5’- AGCTCAGCCAATCCTAGCTC-3’  Reverse: 5’- AATTCCTCGTTGCTGCCTTC-3’  Forward: 5’- ATCACATCTCCCGTCACTCAG-3’  Reverse: 5’- AGTTGCGTGCCTGATTGGATG-3’ |
|  |  |

Primers for cloning

| Gene | Primer Sequence |
| --- | --- |
| Human TOP2A p0.8k | Forward: 5’-CCTCTCGAGTGGAGCAATCACGGTTCAAG-3’ |
|  | Reverse: 5’-TGTAAGCTTCCGCCCGAAGCAGACCAGC-3’ |
| Human SOX9-300ΔC | Forward: 5’-TTCGGATCCATGAATCTCCTGGACCCCTTC-3’ |
|  | Reverse: 5’-GCAGAATTCGTGGCCGTTGGGCGGCAGGTAC-3’ |
