# Supplementary material for: Context-specific role of SOX9 in NF-Y mediated gene regulation in colorectal cancer cells
Source: Nucleic Acids Res. 2015 Jun 3;43(13):6257–69. doi: 10.1093/nar/gkv568 (PMC4513854; doi:10.1093/nar/gkv568)
Supplement: SUPPLEMENTARY DATA [file supp_gkv568_nar-03416-x-2014-File018.docx]

**Supplementary Figure Legends**

**Supplementary Figure S1.** The genomic feature annotation of repetitive sequences of SOX9 peaks. The Sox9 peaks genomic feature annotation (Figure 1A) was expanded by adding 2 additional “features-BED-files” to the analysis program: simpleRepeat.txt (UCSC data base, 962,714 features) and rmsk.txt (UCSC data base, 5,298,130 features = repeat masked regions). The number of SOX9 peaks located in these features was then determined in the same way as for the other genomic features described in the Material and Methods section. The SOX9 peaks are underrepresented in repeat masked regions, while simple repeats have close to random occurrence (311 vs 298 hits). The number of SOX9 peaks located in these features was presented in a log2-transformed bar graph. The graph shows that the SOX9 peaks are underrepresented in repeat masked regions, while simple repeats have close to random occurrence.

**Supplementary Figure S2.** Representative readouts of peaks from SOX9 ChIP-seq (red), NF-YA ChIP-seq (blue), and an input control (black) for *CCNB2* and *CDK1* genes.

**Supplementary Figure S3.** The comparison of three publicly available NF-YA ChIP-seq datasets on cell cycle genes. The screenshots from UCSC Genome Browser website showed the overlapping NF-YA peaks on the promoters of CCNB1, CDK1 and TOP2A genes among different cell lines including GM12878 (B-lymphocyte), K562 and HeLa-S3.

**Supplementary Figure S4. (A)** The peak sequences of SOX9 ChIP-seq with Motif 1 overlap NF-YA ChIP-seq peaks obtained from K562 data base (Encode Accession number: ENCSR000EGR). **(B)** Fifteen representative peaks obtained from K562 data base were verified in HT-29 cells by NF-YA ChIP-qPCR.

**Supplementary Figure S5.** Validation of representative genes identified by SOX9 ChIP-seq (A). Of genes shown in (B), only those with Motif 1 showed enrichment by NF-YA ChIP-qPCR in HT29 cells (B).

**Supplementary Figure S6. (A)** Representative readouts of peaks from SOX9 ChIP-seq (red), NF-YA ChIP-seq (blue), and an input control (black) for *LRIF1* gene. **(B)** To validate the positive *LRIF1* peak in NF-YA ChIP-seqs and negative *LRIF1* peak in SOX9 ChIP-seq, binding of NF-YA and SOX9 in the were confirmed by ChIP-qPCR in HT29 cells using SOX9 and NF-YA antibodies, respectively.

**Supplementary Figure S7.**

pGL3-TOP2A plasmids with wildtype and mutated SOX9 binding sites together with were transfected into HCT116 cells, then luciferase assay was performed 48 hours post-transfection, showing that 1-bp mutation of CCAAT resulted in a reduction of TOP2A promoter activity.

**Supplementary Figure S8.** *In situ* PLA with indicated antibodies demonstrated SOX9–NF-YA interaction in HT29 cells. Nuclei were counterstained with DAPI.

**Supplementary Figure S9.** **The C terminus of SOX9 is responsible for the interaction with NF-YA.** **(A)** Western analysis of HCT116 cell lysates confirmed expression of FLAG-tagged wild-type and mutant SOX9 from indicated constructs. **(B)** Immunofluorescence analysis with anti-FLAG antibody confirmed nuclear localization of mutant FLAG-SOX9ΔHMG-NLS following addition of nuclear localization signal (NLS) to construct FLAG-SOX9ΔHMG in HCT116 cells transfected with the indicated plasmids. FLAG-SOX9-303 ΔC contains two NLSs but lacks the C terminus. **(C)** In comparison to wildtype SOX9, mutated SOX9 with a HMG DNA binding domain did not affect its binding to the promoters of *CCNB1*, *CDK1,* or *TOP2A.*
